# Exploring Instructive Physiological Signaling with the Bioelectric Tissue Simulation Engine

**DOI:** 10.3389/fbioe.2016.00055

**Published:** 2016-07-06

**Authors:** Alexis Pietak, Michael Levin

**Affiliations:** ^1^Allen Discovery Center at Tufts University, Medford, MA, USA

**Keywords:** bioelectric simulation, pattern formation, resting potential, transmembrane voltage

## Abstract

Bioelectric cell properties have been revealed as powerful targets for modulating stem cell function, regenerative response, developmental patterning, and tumor reprograming. Spatio-temporal distributions of endogenous resting potential, ion flows, and electric fields are influenced not only by the genome and external signals but also by their own intrinsic dynamics. Ion channels and electrical synapses (gap junctions) both determine, and are themselves gated by, cellular resting potential. Thus, the origin and progression of bioelectric patterns in multicellular tissues is complex, which hampers the rational control of voltage distributions for biomedical interventions. To improve understanding of these dynamics and facilitate the development of bioelectric pattern control strategies, we developed the BioElectric Tissue Simulation Engine (BETSE), a finite volume method multiphysics simulator, which predicts bioelectric patterns and their spatio-temporal dynamics by modeling ion channel and gap junction activity and tracking changes to the fundamental property of ion concentration. We validate performance of the simulator by matching experimentally obtained data on membrane permeability, ion concentration and resting potential to simulated values, and by demonstrating the expected outcomes for a range of well-known cases, such as predicting the correct transmembrane voltage changes for perturbation of single cell membrane states and environmental ion concentrations, in addition to the development of realistic transepithelial potentials and bioelectric wounding signals. *In silico* experiments reveal factors influencing transmembrane potential are significantly different in gap junction-networked cell clusters with tight junctions, and identify non-linear feedback mechanisms capable of generating strong, emergent, cluster-wide resting potential gradients. The BETSE platform will enable a deep understanding of local and long-range bioelectrical dynamics in tissues, and assist the development of specific interventions to achieve greater control of pattern during morphogenesis and remodeling.

## 1 Introduction

### 1.1 Bioelectricity: Why Model Electrical Activity in Non-Neural Cells?

Explaining and learning to control large-scale pattern is a central unsolved problem, with implications for mitigation of birth defects, and the advancement of regenerative medicine and synthetic bioengineering. The dynamics of signals orchestrating large-scale order *in vivo* are a key area of research, as understanding these signals is an essential first step in developing interventions that alter anatomical outcomes. The dynamics of chemical signals and their gradients are becoming increasingly well-understood (Reingruber and Holcman, 2014; Slack, 2014; Werner et al., 2015). However, endogenous bioelectric signals represent a parallel regulatory system that exerts instructive control over large-scale growth and form. Recent work has demonstrated that ionic and bioelectrical signaling of various cell types underpins a powerful system of biological pattern control [reviewed in Nuccitelli (2003a), McCaig et al. (2005), Levin (2012, 2014), Levin and Stephenson (2012), and Tseng and Levin (2013)]. Importantly, endogenous bioelectric gradients across tissues can be a very early pre-pattern for subsequent transcriptional and morphogenetic events. For example, during craniofacial development of frogs, specific transmembrane voltage (V_mem_) patterns determine the downstream shape changes and gene expression domains of the developing face (Vandenberg et al., 2011; Adams et al., 2016) and brain (Pai et al., 2015). Furthermore, experimental modulation of cell V_mem_ states can radically alter large-scale anatomy, for example, inducing eye formation in ectopic body areas, such as the gut, where the master eye regulator Pax6 cannot induce eyes (Pai et al., 2012), reprograming the regeneration blastemas of planaria to produce heads instead of tails (Beane et al., 2011), or rescuing normal brain patterning despite the presence of mutated neurogenesis genes, such as Notch (Pai et al., 2015).

### 1.2 Local and Long-Range Order in Bioelectrical Networks

On the scale of single cells, the V_mem_ spanning every living cell’s plasma membrane is a demonstrated regulator of key processes, such as cell proliferation (Blackiston et al., 2009), programed cell death (Boutillier et al., 1999; Wang et al., 1999), and differentiation (Ng et al., 2010), and is known to be a factor in the activation of immune cells (Bronstein-Sitton, 2004). For example, despite the action of growth factors, stem cells have been inhibited from differentiation by preventing the cells from developing a hyperpolarized V_mem_ (Sundelacruz et al., 2008). The bioelectric properties of single cells are fairly well-understood (Lodish et al., 2000; Wright, 2004). However, bioelectric states often regulate large-scale anatomical properties, such as axial polarity (Marsh and Beams, 1952; Beane et al., 2011), organ size (Perathoner et al., 2014) and shape (Beane et al., 2013), and induction of formation of whole appendages (Adams et al., 2007; Tseng et al., 2010). Moreover, pattern control involves long-range coordination of bioelectric states. In metastatic conversion (Morokuma et al., 2008; Blackiston et al., 2011; Lobikin et al., 2012), tumor suppression (Chernet and Levin, 2014; Chernet et al., 2015), brain size regulation (Pai et al., 2015), and head–tail polarity in planarian regeneration (Beane et al., 2011), the patterning outcome in one region of the animal is a function of the bioelectric states of both local and remote cells. Thus, it is imperative to understand not only how ion channel and pump activity controls single-cell electrical properties but also how electrical gradients self-organize, propagate, and evolve in multicellular networks. Moreover, understanding the origin of developmental order also requires that we understand how tissue-level gradients of bioelectric properties arise.

In a multicellular collective, endogenous patterns of V_mem_ and electric fields provide positional information and achieve long-range coordination of cell activity. As in the central nervous system, this occurs because cells in a tissue are not isolated, but are electrochemically connected (and, therefore, communicating) in several ways, including intracellular channels known as gap junctions [GJ (Goodenough and Paul, 2009)], and by ephaptic coupling created by local field potentials, which enable one cell’s V_mem_ activity to influence that of its neighbor’s (Zhou et al., 2012). These connections between cells create bioelectrical circuits involving long-range signal patterns through whole structures, which have been determined crucial for developing embryos (Jaffe, 1981; Hotary and Robinson, 1990; Hotary and Robertson, 1994; Shi and Borgens, 1995), normal limb development of animals (Altizer et al., 2001), healing of wounds (Nuccitelli, 1992, 2003a; McCaig et al., 2005; Zhao, 2009), and even in continuous tumor suppression in adult animals (Chernet and Levin, 2013, 2014). The ability for cells to couple and communicate makes local changes to cell V_mem_ relevant in terms of long-range signals capable of affecting the whole. Likewise, the *inability* for cells to form communication networks, for instance, due to improper expression or function of GJ connections, is observed in disease processes, such as cancer (Leithe et al., 2006; Trosko, 2007). Even briefly altering the bioelectric connectivity of a cellular network enables rewriting of an organism’s target morphology. For example, genomically normal fragments of planarian flatworms can be induced to regenerate heads with shapes and internal anatomy belonging to other extant species (Emmons-Bell et al., 2015), or changed to a two-headed form that regenerates with two heads in perpetuity, illustrating the ability to stably re-wire bioelectric circuits with permanent changes to the overall anatomy (Oviedo et al., 2010).

Another important bioelectrical signal relevant to multicellular clusters is a voltage gradient known as the trans-epithelial potential (TEP), which forms at the outer boundary of an organ or organism. The TEP is also implicated in normal developmental processes (Shi and Borgens, 1995), wound healing (Zhao, 2009), and disease processes, such as cystic fibrosis (Hay and Geddes, 1985), fungal infection (Gow and Morris, 1995), inflammation, and cancer (Soler et al., 1999). The TEP is created when multicellular structures develop impermeable tight junctions (TJ) between cells at the exterior boundary (Hay and Geddes, 1985); disruptions to this process induce electric fields that serve as guidance cues for many migratory cell types during injury response (McCaig, 1990; Zhao, 2009; Yamashita, 2013) and limb development (Borgens, 1984; Borgens et al., 1987). Understanding plasma membrane voltage gradients and transepithelial potentials, and their spatio-temporal transitions *in vivo*, is a key enabling step for the field of developmental bioelectricity and its applications.

### 1.3 Modeling: The Need for *In Silico* Simulation

Understanding and learning to control patterning signals requires a quantitative appreciation of their intrinsic dynamics and the way they evolve through time. Since the pioneering work of Turing (Turing, 1952; Raspopovic et al., 2014; Watanabe and Kondo, 2015), much effort has gone into mathematical modeling of the dynamics of biochemical signals and their gradients. While there are many platforms for modeling spiking activity in the brain (Bower and Beeman, 2007), there are few available frameworks for formulating predictive models of bioelectric signaling during slower processes involved in somatic cell pattern regulation (Cervera et al., 2016), and even fewer working from the more biorealistic perspective of ionic concentrations and movements, rather than an equivalent electric circuit model. Such biorealistic models are crucial if we are to develop effective interventions that target powerful bioelectric control processes. Furthermore, ion channels and GJs are themselves voltage-sensitive (Nau, 2008; Palacios-Prado and Bukauskas, 2009). This means that cell groups can implement highly non-linear behaviors and feedback loops that are too complex to predict or control by direct inspection. While recent efforts have begun to model some of the interesting behavior of these GJ-coupled dynamical systems (Cervera et al., 2014, 2015; Law and Levin, 2015), there is a need for a flexible, powerful platform to facilitate *in silico* experimentation and model-building, and for connecting bioelectric dynamics with other aspects of physiology, physical forces, and genetic networks. The availability of a realistic modeling system for bioelectricity will enable (1) formulation of models of specific patterning events based on realistic physiological and channel expression data, (2) design of predicted intervention strategies for inducing desired changes in electrical state and downstream patterning outcomes, and (3) investigation of the broader capabilities of non-neural bioelectrical networks for use in synthetic biology (Doursat and Sanchez, 2014; Kamm and Bashir, 2014; Mustard and Levin, 2014) and unconventional computation architectures (Adamatzky and Jones, 2011; Adamatzky et al., 2012).

As a core component of enabling the unraveling of the bioelectrical dynamics of tissues in this exciting emerging field, we have created the Bio-Electric Tissue Simulation Engine (BETSE) to quantitatively explore bioelectrical signals in networked cell collectives. BETSE integrates a diverse range of mechanisms and physiologies to enable model building and hypothesis testing at a level congruent with experimental observables, including electrodiffusion of multiple ions under chemical and electrical gradients in various contexts; consideration of concentration, charge, voltage, and current in both intra- and extracellular networks in order to capture important signals, such as tissue-wide endogenous ion currents, TEP, and local field potentials; and dynamic control of membrane permeability and gap junction state to simulate voltage and ligand-gated channels. This work is the first in a series of studies modeling specific patterning systems, and using BETSE to infer targeted modulation strategies. Here, we discuss the design of BETSE, validate BETSE’s bioelectrical modeling performance, and provide some insights into the fundamental mechanisms involved in patterning of networked multicellular clusters.

## 2 Materials and Methods

### 2.1 Model Overview

Whether working with metals, semiconductors, or the salt-water electrolyte of biological systems, voltages (electric potential energies) are created by net electrical charge. In typical electrical systems, such as metals and semiconductors, the charge carriers are electrons or the absence of electrons (holes). In electrolytes, ions from dissolved salts can develop concentration profiles generating net charge in a region of space and, therefore, create voltages. Furthermore, mass flux of ions can generate a net current, which is associated with intracellular and tissue-wide electric fields. Therefore, ions are the fundamental units of the bioelectrical system, and their concentrations, mass fluxes, and transport mechanisms are ultimately important. BETSE can consider ions relevant to most living systems: Na^+^, K^+^, Cl^−^, Ca^2+^, ${\text{HCO}}_{3}^{-}$, H^+^, and charged macromolecules, such as proteins (X^−^). In addition, BETSE can consider the movement of a charged biomolecule, such as a voltage reporter dye, glutamate, serotonin or inositol triphosphate (symbolized as Y^n−^ or Y^n+^, where n is a variable charge number) present at low concentrations and, therefore, assumed to not affect voltage directly due to its inconsequential contribution to local charge density.

Cells create and control V_mem_ by selectively altering ion fluxes across their membrane. Ion pumps, such as the sodium potassium pump (Na/K-ATPase), use free-energy released from ATP hydrolysis to move ions across the insulating cell membrane, creating net ionic charge density and voltage gradients inside and outside of the cell, similar to a self-charging capacitor (Veech et al., 1995). Ion channels in the plasma membrane allow charge to move under these concentration and voltage gradients, altering charge densities and thereby changing the concentration and voltage gradients to create bioelectrical signals. At its core, BETSE keeps track of ion concentrations and ion fluxes in space and time, reducing them to net charge distributions inside and outside of the cell, using these net charges to calculate voltages inside and outside of the cellular space, calculating changes to concentrations resulting from ion mass fluxes resulting from concentration/voltage gradients and by active ion pumps, and calculating endogenous currents from the net mass flux of ions. Membrane permeability to specific ions is used as a dynamic variable to simulate the action of specific ion channels (including K^+^ leak channels, calcium gated K^+^ and Cl^−^ channels, and voltage-gated Na^+^, K^+^, and Ca^2+^ channels).

**Figure 1:**
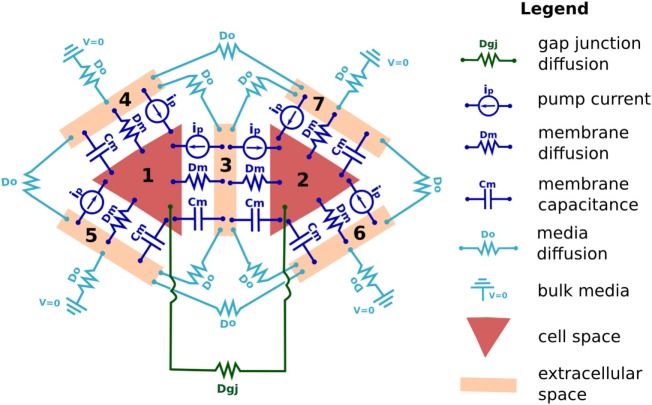
**The fundamental “bioelectrical circuit” implemented in BETSE, shown on a simplified geometry of two triangular cells (1 and 2) surrounded by their respective extracellular spaces (3–7)**. Note that in BETSE, and in contrast to the simplified image shown, cells are defined from a Voronoi diagram and are polygonal with four or more membranes, and that a larger network of 10–1000 cells is considered in simulations. Each cell–extracellular junction has a capacitive component (membrane capacitance *C_m_*), a “resistive” component (cell membrane diffusion coefficients, *D_m_*), and a variable current source (representing the action of pumps, *i_p_*). Transfer between two cells occurs via GJ, which are represented by a “resistive” component (*D_gj_*). Transfer between extracellular spaces and to the environment is handled using “resistive” components (*D_o_*). Boundary conditions at the global environmental boundary are represented by grounded voltage (*V*  = 0) and fixed concentrations representing an open boundary with Dirichlet conditions. Self-capacitances for each cell and extracellular space are not shown.

The following details how electro-diffusive transport, voltage calculations, ion pumps, ion channel dynamics, voltage-sensitive GJ, and electroosmotic flows are handled in BETSE. Further details regarding BETSE’s underlying theory and implementation can be found in Supplementary Material. Table 1 summarizes key parameter and typical variable values and their units. A highly simplified schematic of the “bioelectric circuit” implemented in BETSE is shown in Figure 1.

**Table 1:** **Main model parameters and variables**.

Parameter	Description	Typical value	Units
*i*	Ion index (*i* = *Na, K, Cl, Ca, H, M*)		
${\text{D}}_{{\text{o}}_{\text{i}}}$	Free diffusion coefficient for ion *i*	1.0 × 10^−9^	$\frac{{\text{m}}^{\text{2}}}{\text{s}}$
*t*	Time	10	s
*x, y*	Spatial coordinates	500	*μ*m
*h*	System height	10	*μ*m
$\Delta {\text{G}}_{\text{ATP}}^{\text{o}}$	Standard free energy of ATP hydrolysis	37	kJ/mol
*T*	Temperature	310	K
*F*	Faraday’s constant	96,485	C/mol
*R*	Ideal gas constant	8.3145	J/K mol
*q*	Electron charge constant	1.6 × 10^−19^	C/ion
*k_b_*	Boltzmann constant	1.38 × 10^−23^	J/K
*v_cell_, v_ecm_*	Cell and extracellular volume	7.85 × 10^−16^	m^3^
σ*_cell_*, σ*_mem_*, σ*_ecm_*	Cell, membrane and extracellular surface area	3.14 × 10^−10^	m^2^
*c_mem_*	Membrane capacitance	0.022	F/m^2^
*c_self_*	Electrolyte-induced self-capacitance	0.86	F/m^2^
*α_pump_*	Maximum rate constant for pump	2.0 × 10^−7^	1/s m^2^
ν_1/2_*_GJ_*	GJ voltage-gating half-closed parameter	15	mV
*d_mem_*	Cell membrane thickness	7.5 × 10^−9^	m
*d_gj_*	Intercellular spacing	26.0 × 10^−9^	m
*μ*	Water viscosity	5.0 × 10^−3^	Pas

**Variable**	**Description**	**Typical value**	**Units**

${\text{c}}_{{\text{i}}_{\text{ext}}}$	Extracellular concentration	1 to 150	$\frac{\text{mol}}{{\text{m}}^{\text{3}}}$
${\text{c}}_{{\text{i}}_{\text{int}}}$	Intracellular concentration	1 to 150	$\frac{\text{mol}}{{\text{m}}^{\text{3}}}$
${\text{D}}_{{\text{mem}}_{\text{i}}}$	Membrane diffusion coefficient for ion *I*	1.0 × 10^−18^	$\frac{{\text{m}}^{\text{2}}}{\text{s}}$
${\text{P}}_{{\text{mem}}_{\text{i}}}$	Membrane permeability for ion *i*	0.13	$\frac{\text{nm}}{\text{s}}$
*V_cell_, V_env_*	Voltage in cell and environment	−10 to −80	mV
*V_mem_*	Transmembrane voltage	−10 to −80	mV
${\vec{\Phi }}_{\text{i}}$	Mass flux of ion *i*	1.0	$\frac{\mu \text{mol}}{\text{s}{\text{m}}^{\text{2}}}$
*ρ_e_*	Ionic charge density	600	$\frac{C}{{\text{m}}^{\text{3}}}$
$\vec{\text{J}}$	Ionic current density	10–500	$\frac{\mu \text{A}}{{\text{cm}}^{\text{2}}}$
$\beta_{\text{GJ}}^{\text{o}}$	GJ diffusion scaling-coefficient	5.0 × 10^−7^	
*β_TJ_*	TJ diffusion scaling-coefficient	1.0 × 10^−7^	
*D_NaV_, D_KV_*	Max membrane diffusion for voltage-gated channel	1.0 × 10^−14^	$\frac{{\text{m}}^{\text{2}}}{\text{s}}$
$\vec{\text{E}}$	Electric field	1 × 10^5^	V/m

### 2.2 BETSE Platform and Performance

BETSE is a finite volume method multiphysics simulation platform, uniquely specialized to work with a range of bioelectric phenomena arising in biological tissues, which are highly spatially heterogeneous by nature.

BETSE was implemented in Python 3.4, making heavy use of the scientific and engineering toolboxes Numpy, Scipy, and Matplotlib (Millman and Aivazis, 2011).

To make each time step of a simulation as quick as possible, BETSE uses matrix-based differential equation solvers, making memory one of the limitations of simulation size and extent. Simulating a square millimeter of tissue (~10,000 cells) with all features enabled (e.g., extracellular space simulation, electroosmotic fluid flow, all ion types included) uses approximately 14 Gb of RAM, and is considered the current limit of simulation size.

BETSE code is available from the public repository: http://ase.tufts.edu/biology/labs/levin/resources/software.htm

### 2.3 Core Mathematical Strategy

**Figure 2:**
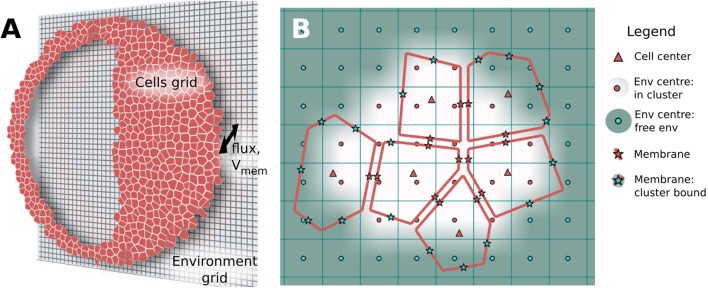
**BETSE computations use an irregular Voronoi-based cell grid interacting with a regular square environment grid to model heterogeneous tissues (A)**. The cell grid is composed of cell center (“Δ”) and membrane points (“*”) **(B)**. Membranes that lack a neighboring cell (and, therefore, interact only with the global environment) are identified using a boundary search algorithm. The environmental grid consists of regularly spaced points (“*o*”) which are tagged as being internal or external to the cell cluster **(B)**. The intercellular spaces of the cell grid are assumed to be connected to the extracellular environment by fluxes or gradients **(A)**, which use points interpolated between the cell membrane and environmental grid midpoints with a weighting function (cell membranes seen per grid square) to properly assign the mole transfer for a mass flux between cell and environment **(B)**.

Biological tissue represents a challenging modeling scenario due to its highly heterogeneous nature, where closely spaced (~10–30 nm), membrane bound, electrolyte-filled cells are individually interacting with a small extracellular space at individual plasma membranes, and where the extracellular spaces connect with a continuous, aqueous environment at the cell cluster boundary. Individual cells are also connected internally via transmembrane channels, such as GJ, which enable passage of small molecules and ionic current between cells. To manage this involved biophysical situation, BETSE uses an irregular Voronoi diagram-based cell grid, embedded within a regular square environmental grid, to model the heterogeneous nature of tissues, while also allowing modeling of a continuous environmental space around the cell cluster (Figure 2A).

Each modeled cell in the cell grid has a center point (indicated in grid diagrams as Δ, see Figure 2), where scalar cell properties, such as concentration (*c_i_*) and intracellular voltage (*V_cell_*) are defined. Each cell also has a unique volume (*vol_cell_*) and perimeter representing the cell membrane. This allows unique membrane properties, such as *V_mem_*, to be defined for each segment of each individual cell membrane, thereby opening the possibility for study of individual cell polarizations and self-electrophoresis/electroosmosis of membrane-bound ion pumps and channels (Jaffe, 1981; McLaughlin and Poo, 1981).

Membrane-specific scalar and vector properties are defined at each membrane segment midpoint (indicated as * in Figure 2B). Each membrane segment also has normal and tangent unit vectors. The membrane midpoints of each cell interface with the central points of local environmental grid squares (red “o” in Figure 2) via a nearest-neighbor interpolation scheme. A weighting function (cell membranes seen per grid square) is used to properly assign the mole transfer for a mass flux between cell and environment, thereby conserving mass and charge of the system (see Supplementary Material for more information).

The interconnected grid systems of BETSE, which models individual cells as discrete patches, make it possible to shape the cluster into complex forms and to cut holes into the cell cluster (before or during a simulation). Holes in the tissue represent the continuous electrolyte in the region of the hole. This enables study of simple vasculature (e.g., capillaries feeding the tissue by diffusion from the environment), cysts (such as the model shown in Figure 2A), and wounding. BETSE uses bitmaps to define the shape of the cluster, cut holes, and to assign specific properties (i.e., membrane permeability) to desired regions of the modeled tissue (see Supplementary Material).

The core mathematical operators of differential equations used in BETSE are:

- *gradient* (∇*s_j_*), which calculates the degree of change in the spatial property over space at grid point j
- *divergence* ($\nabla \cdot {\vec{F}}_{j}$), which measures the amount of outward flow of a vector field from each point in space, measuring the presence of a source (+ divergence) or sink (− divergence) at grid point j, and
- the *Laplacian* (∇^2^*s_j_* = ∇ ⋅ ∇*s_j_*), which is most intuitively expressed as the divergence of the gradient of a scalar property. When discretized, the Laplacian is a matrix, which can be inverted to give the inverse of the operation, such that if ∇^2^*S_j_* = *c_j_* then *S_j_* = ∇^−2^*c_j_*.

Discrete versions of gradient, divergence, and Laplacian/inverse Laplacian were defined, using standard finite difference and finite volume techniques (Schafer, 2006), on the cell and environmental grids. These core mathematical operators were then used where required in specific differential equation expressions.

The detailed features of the cell and environmental grids, the specific definition of the above mathematical operators on each of the grids, and the interaction between the cell and environmental grid models, are discussed in Supplementary Material.

### 2.4 Bio-Electrochemical Mass Transport

Ion transport in bioelectrical systems is influenced by gradients of both concentration (∇*c_i_*) and voltage (∇*V*), with ions passively moving by a process known as *electrodiffusion* – a combination of regular diffusion and electrophoretic transport. In general, electrodiffusion is described by the Nernst–Planck differential equation, describing the rate of change in the concentration *c_i_* of an ion *i* with charge *z_i_* and diffusion constant *D_i_*:

(1) $$\frac{dc_{i}}{\mathrm{dt}}=\nabla \cdot \left(D_{i}\nabla c_{i}+\frac{D_{i}z_{i}q}{k_{b}T}\nabla V-\vec{u}c_{i}\right)$$

here, $\vec{u}$ is a fluid flow [e.g., electroosmotic flow field (Andreev, 2013)], *q* is the electron charge constant, *k_b_* is the Boltzmann constant, and *T* is the temperature (see Table 1).

Ions are actively transported by pumps in the cell membrane. Both passive and active transport processes generate ion fluxes (Φ*_i_*). These combined fluxes can lead to changes in concentration and charge density, and can generate a system-wide ionic current density $\vec{J}$.

**Figure 3:**
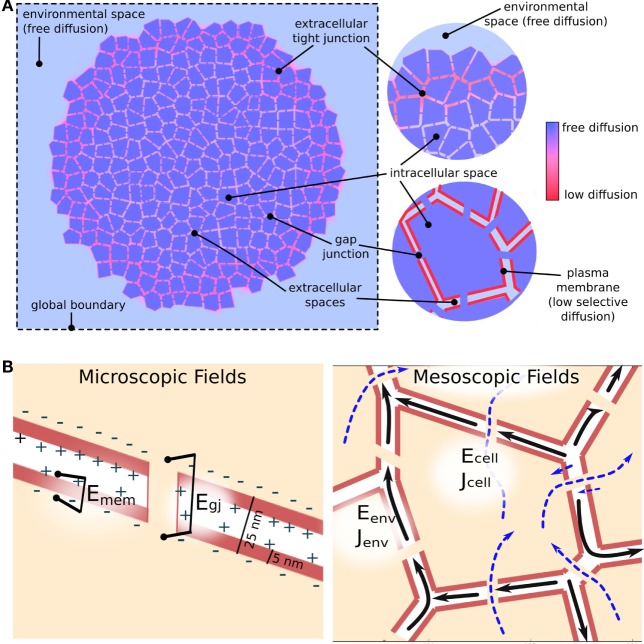
**Electrodiffusive mass transport in a GJ networked cell cluster is assumed to follow three pathways (A): (1) transmembrane – between intra- and extracellular spaces across the plasma membrane; (2) inter-cellular – between cellular spaces via GJ; and (3) environmental – between extracellular spaces and in the global environment**. The degree of movement of ions in both chemical and electrical gradients is handled using spatially varying diffusion coefficients, which reduce from free-diffusion coefficients in the environmental space to minimal values across simulated tight junctions and plasma membranes **(A)**. In addition to concentration gradients, ions are assumed to move under the influence of voltage gradients [electric fields **(B)**]. Strong electric fields are assumed to exist on a microscopic scale across membranes and gap junctions due to heterogeneous charge distribution at membrane interfaces separated by tens of nanometers [**(B)**, Microscopic fields]. Weaker, mesoscopic scale (10–100 *μ*m) electric fields are assumed to be generated by net ion currents in the intra- and extracellular spaces [**(B)**, Mesoscopic fields].

BETSE assumes passive electrodiffusive mass transport in a multicellular cluster follows three distinct pathways: (1) *transmembrane*, via intra- and extracellular spaces across the plasma membrane; (2) *intercellular*, between cellular spaces via gap junctions; and (3) e*xtracellular*, between extracellular spaces and within the global environment (Figure 3). Active transport from ion pumps is always assumed to be transmembrane. Therefore, BETSE considers the following sources of ion flux for an ion *i*:

- *Transmembrane*, from passive electrodiffusive transport resulting from gradients between the local intra- and extracellular spaces using the Goldman–Hodgkin–Katz Flux equation (GHK Flux equation), which is derived from the Nernst–Planck Differential equation for the case of electrodiffusion across a cell membrane for a non-steady-state V_mem_ (Bowman and Baglioni, 1984):
  (2) $$\Phi_{{\mathrm{mem}}_{i}}=\frac{z_{i}V_{\mathrm{mem}}FD_{i}}{{\mathrm{R T d}}_{\mathrm{mem}}}\left(\frac{c_{{\mathrm{cell}}_{i}}-c_{{\mathrm{env}}_{i}}\exp\left(-\frac{z_{i}V_{\mathrm{mem}}F}{\mathrm{RT}}\right)}{1-\exp\left(-\frac{z_{i}V_{\mathrm{mem}}F}{\mathrm{RT}}\right)}\right)$$
  here, *F* is the Faraday constant, *R* is the ideal gas constant, and *d_mem_* is the plasma membrane thickness (see Table 1). Positive fluxes are directing mass into cells.
- *Transmembrane*, from active transport resulting from ion pump activity. Details of how the dynamic ion pump rates (α) are calculated are given in section 2.7:
  (3) $$\Phi_{{\mathrm{pump}}_{i}}=\alpha \left(c_{{\mathrm{cell}}_{i}},c_{{\mathrm{env}}_{i}},V_{\mathrm{mem}},t\right)$$
  Positive fluxes are directing mass into cells.
- *Intercellular*, from passive electrodiffusive transport resulting from gradients between neighboring, GJ networked cells:
  (4) $${\vec{\Phi }}_{{\mathrm{cell}}_{i}}=-D_{i}\nabla c_{{\mathrm{cell}}_{i}}-\frac{D_{i}z_{i}q}{k_{b}T}c_{{\mathrm{cell}}_{i}}\nabla V_{\mathrm{cell}}+{\vec{u}}_{\mathrm{cell}}c_{{\mathrm{cell}}_{i}}$$
- *Extracellular*, from passive electrodiffusive transport resulting from gradients between neighboring environmental spaces:
  (5) $${\vec{\Phi }}_{{\mathrm{env}}_{i}}=-D_{i}\nabla c_{{\mathrm{env}}_{i}}-\frac{D_{i}z_{i}q}{k_{b}T}c_{{\mathrm{env}}_{i}}\nabla V_{\mathrm{env}}+{û}_{\mathrm{env}}c_{{\mathrm{env}}_{i}}$$

Changes in concentration are made by assuming the concentration change in an ion *i* depends on the divergence of the net ($\Phi_{{\mathrm{tot}}_{i}}$) sum of all fluxes of the ion entering or changing in a particular region of the space (i.e., cells or environment):

(6) $$\frac{\partial c_{i}}{\mathrm{\partial t}}=-\nabla \cdot \Phi_{{\mathrm{tot}}_{i}}$$

Net ionic charge density was calculated by summing all ion concentrations at a region of space:

(7) $$\rho_{e}=\underset{i}{\sum }Fz_{i}c_{i}$$

The dynamics of ionic charge density were calculated from the mass flux of all ions:

(8) $$\frac{\partial \rho_{e}}{\mathrm{\partial t}}=\underset{i}{\sum }Fz_{i}\Phi_{i}$$

The total current density of the environment or cell, $\vec{J}$, was calculated using the continuity equation in combination with the assumption of bulk electro-neutrality for electrolytes due to charge screening. Using the Continuity equation for current, the current density in a region follows:

(9) $$\nabla \cdot {\vec{J}}_{o}+\frac{\partial \rho_{e}}{\mathrm{\partial t}}=0$$

As electro-neutrality (zero net charge density) must be preserved in the bulk electrolyte, the base current density calculated by BETSE (${\vec{J}}_{o}$) was corrected by assuming that an internal electric field develops in the bulk electrolyte as a result of charge screening, which is the negative gradient of an electric potential *φ_int_*:

(10) $$\vec{J}=J_{o}-\nabla \psi_{\mathrm{int}}$$

Substituting equation (10) into equation (9) and rearranging to solve for the internal electric potential:

(11) $$\psi_{\mathrm{int}}=\nabla^{-2}\left(\nabla \cdot {\vec{J}}_{o}+\frac{\partial \rho_{e}}{\mathrm{\partial t}}\right)$$

After obtaining *φ_int_*, it is used with equation (10) to produce the corrected current density for the system. Current density in the environment and in cell spaces was treated as separate.

Note that as movement in both concentration and electrical gradients can occur, the transport properties of bioelectrical systems cannot be strictly reduced to electrical constants, such as resistance or conductance. However, examining the Nernst–Planck equation [equation (1)] reveals that the diffusion coefficient *D* is able to serve as the constant of proportionality for movement in both chemical and electrical terms. In the absence of a concentration gradient, and multiplying by *F z* to convert mass flux to ionic current density, the Nernst–Planck Flux equation reduces to:

(12) $${\vec{J}}_{i}=-\frac{FD_{i}z^{2}q}{k_{b}T}c_{i}\nabla V$$

Noting that the definition of an electric field is $\vec{E}=-\nabla V$, equation (12) parallels the equation relating current density to electric field via media conductivity $\frac{1}{\gamma }$:

(13) $$\vec{J}=\frac{1}{\gamma }\vec{E}$$

Therefore, BETSE makes use of diffusion constants to characterize ion transport in different regions of the multicellular cluster, but can approximate conversions between conductivity and the diffusion constant.

Note that for movement across a membrane with thickness *d_mem_*, the permeability of the membrane is simply *P_mem_* = *D_mem_*/*d_mem_*.

### 2.5 Biological Voltages

#### 2.5.1 Bioelectric Voltage Calculations Using a Maxwell Capacitance Matrix

The Poisson equation ($V=-\frac{\rho_{e}}{\varepsilon }$, where *ρ_e_* is electronic charge density and ε is medium electrical permittivity) is typically used to determine voltage from charge density. In air, a charged object will emanate a voltage gradient (electric field) into the space around it according to the Poisson equation. However, electrolytes are more complex. Due to the presence of mobile, oppositely charged ions in electrolytes, objects with steady-state voltages or bound charges collect an opposite surface charge from the electrolyte to form an electrical double layer approximately 1-nm thick in biological systems, which screens the voltage/charge of the object and a prevents long-range electrical field from developing at macroscopic distances into the electrolyte (Bazant and Squires, 2004). Moreover, biological systems are highly heterogeneous, with opposite-sign charge distributed at intra- and extracellular interfaces of the plasma membrane. This means opposite sign charges are separated by the small membrane thickness (3.5–9 nm), and that in a collective of many cells with closely interfacing membranes, charges are present in the low-volume extracellular space that is approximately 5–50-nm wide between cells. Therefore, a new technique was adopted to model voltage in the biological tissue. Voltages in the intra- and extracelluar spaces (*V_intra_, V_extra_*), and the related *V_mem_* = *V_intra_* − *V_extra_*, were calculated from net ionic surface charge distributions using a formulation called the *Maxwell Capacitance Matrix* (Clements et al., 1975; Heinzel, 2008).

**Figure 4:**
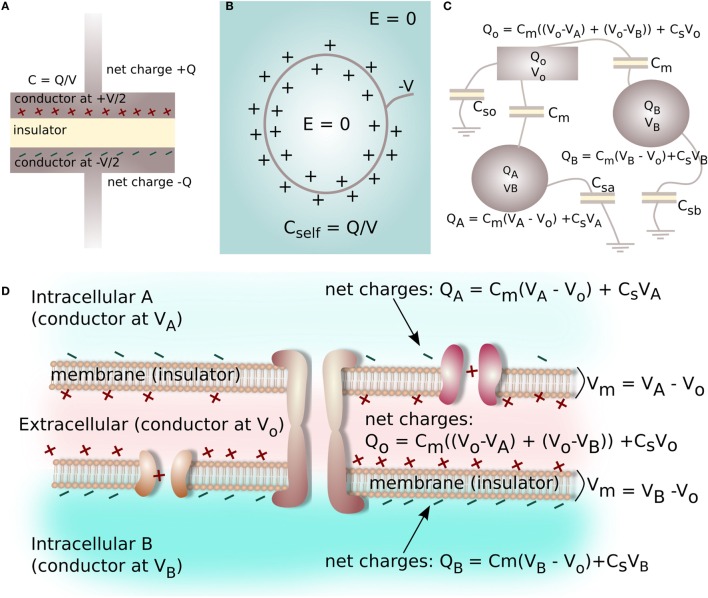
**A capacitor is a device commonly characterized by two metal plates (conductors) with equal and opposite charge Q on either plate, separated by an insulating layer (A)**. A surface at a static negative voltage V, submerged in an aqueous electrolyte, does not produce a long-range electrical field (voltage gradient) in the electrolyte due to charge screening and the formation of the electrical double layer **(B)**. The ratio of screening charge Q to surface voltage V is a self-capacitance for the system **(B)**. Multiple insulator-separated conductors with variable amounts of charge and voltage on each conductor form a capacitive system, with the relationship between charge and voltage on the conductors described by a Maxwell Capacitance Matrix **(C)**. Likewise, the interface between two cells can be reduced to a capacitive electrical system consisting of three conductive spaces with net charge and voltage (2 intracellular, 1 extracellular) separated by two insulators (2 cell membranes, capacitance C_mem_), where each electrolyte-filled space has a self-capacitance related to electrolyte screening **(D)**.

Capacitance is typically known in terms of an electrical device characterized by two metal plates (electrical conductors) with equal and opposite charge (±*Q*) on either plate, which are separated by a layer of insulating material (Figure 4A). The capacitance (*C*) is defined by the ratio of the voltage (*V*) between the plates in relation to the charge *Q* on each plate (Figure 4A):

(14) $$C=\frac{Q}{V}$$

However, arrangements of conductors can involve capacitance via multiple insulator-separated conductors with variable amounts of charge and voltage on each conductor (Figure 4B). For the case of multiple conductors, the basic capacitance relation shown in equation (4) must be extended to parameterize more complex arrangements. For instance, for the three conductors shown in Figure 4C, the charge *Q* on each conductor can be related to voltages and capacitances of the system as:

(15) $$\begin{array}{l} \begin{array}{rl} Q_{A} & =C_{m}\left(V_{A}-V_{o}\right)+C_{\mathrm{sa}}V_{A}\\ Q_{B} & =C_{m}\left(V_{B}-V_{o}\right)+C_{\mathrm{sb}}V_{B}\\ Q_{o} & =C_{m}\left(V_{o}-V_{A}\right)+C_{m}\left(V_{o}-V_{B}\right)+C_{\mathrm{so}}V_{o} \end{array} \end{array}$$

here, *C_m_* is a capacitance connecting the conductors, and *C_sa_, C_sb_*, and *C_so_* are the self-capacitances of the conducting objects.

The self-capacitance of a conductor describes how much charge is acquired on the conductor/unit voltage applied to its surface. For electrolytes, self-capacitance is related to the ability of the electrolyte to screen voltage on a submerged object (see Figure 4B). Using Boltzmann relations for low voltages, in an electrolyte the ionic charge density *ρ_e_* forming near the surface of an object with a surface voltage *φ_o_* can be expressed as a function of distance, *x*, away from the surface as:

(16) $$\rho_{e}\left(x\right)=-\varepsilon_{o}\varepsilon_{r}\kappa^{2}\varphi_{o}\exp\left(-\kappa x\right)$$

where κ is the inverse Debye length for the electrolyte, which assuming the approximation for a symmetric monovalent electrolyte with total molar concentration *C_tot_* for a typical BETSE system is expressed:

(17) $$\kappa =\sqrt{\frac{2F^{2}C_{\mathrm{tot}}}{\varepsilon_{r}\varepsilon_{o}RT}}=1.2x10^{9}\;\frac{1}{m}$$

Integrating equation (16) with respect to *x* from the surface at 0 to infinity, and dividing by the surface voltage to approximate a self-capacitance/unit surface area for surfaces in the electrolyte:

(18) $$c_{\mathrm{self}}=\frac{\rho_{e}}{\varphi_{o}}=\varepsilon \varepsilon_{r}\kappa =0.86\;\frac{F}{m^{2}}$$

The system of linear equations derived when considering more complex arrangements of insulator-separated conductors can be expressed in a matrix form (Heinzel, 2008). For the highly simplified system shown in Figure 4B, and described by equation (15), the Maxwell Capacitance matrix (**M**) interrelating charge *Q_k_* and voltage *V_k_* on each conductor is:

(19) $$\begin{array}{rl} \left[\begin{array}{c} Q_{A}\\ Q_{B}\\ Q_{o} \end{array}\right] & =\left[\begin{array}{c} \begin{array}{ccc} C_{m}+C_{\mathrm{sa}} & 0 & -C_{m}\\ 0 & C_{m}+C_{\mathrm{sb}} & -C_{m}\\ -C_{m} & -C_{m} & 2C_{m}+C_{\mathrm{so}} \end{array} \end{array}\right]\left[\begin{array}{c} V_{A}\\ V_{B}\\ V_{o} \end{array}\right]\\ \bar{Q} & =M\bar{V}\\ \bar{V} & =M_{\mathrm{inv}}\bar{Q} \end{array}$$

For the case where the set of charges $\bar{Q}$ are known, the corresponding set of voltages $\bar{V}$ can be found by calculating the pseudo-inverse of the Maxwell Capacitance matrix (**M***_inv_*) using a singular-value decomposition method.

In the biological system, we propose every point of contact between two cells represents a situation similar to the one shown in Figure 4C. In order to calculate voltage within the closely-spaced intra- and extracellular regions, and to thereby derive V_mem_ for a cell cluster, each cell–cell interface is reduced to a capacitive electrical system consisting of three conductors: two intracellular spaces and one extracellular space, which are each separated by two cell membranes with capacitance *C_mem_*. Each space has net ionic charge *Q_k_* and voltage *V_k_*. The self-capacitance of each space is related by equation (18). This allows a Maxwell Capacitance Matrix identical to the one defined in equation (19) to be constructed for a single cell–cell junction (Figure 4D).

In BETSE, a typical cell cluster consists of many hundred to thousand cell–cell interfaces and, therefore, has a very large **M**, the pseudo-inverse of which was used to calculate voltage in each intra- and extracellular space from net charge *Q_k_* in each region. To complete the calculation, V_mem_ are calculated by taking the difference between the intra- and extracellular voltages at a respective membrane point.

Note that the use of the Maxwell Capacitance Matrix to derive V_mem_ is only one component of the computation of bioelectrical variables – a simplified bioelectrical “circuit” is shown in Figure 1, and must also include electrodiffusive transport of ions via transmembrane, intercellular, and extracellular networks, in addition to active transport of ions by pumps, as described in section 2.4.

#### 2.5.2 Assumptions Regarding the Biological Electric Field

It is important to clarify that while it is well known that the ions of electrolytes screen voltages arising from static charge distributions, thereby preventing electric field (voltage gradient) from *static* charge distributions from being seen past the electrical double layer, any net ionic current density ($\vec{J}$) arising from ion fluxes in the biological system is known to generate a small magnitude observable macroscopic electric field (${\vec{E}}_{\mathrm{global}}$) according to:

(20) $${\vec{E}}_{\mathrm{global}}=\gamma \vec{J}$$

where γ represents the media resistivity of approximately 0.02 Ωm. These endogenous currents and related global electric fields have been observed directly and are on the magnitude from 1 to 1000 $\frac{\mu \text{A}}{{\text{cm}}^{\text{2}}}$ and exist in the extra- and intracellular spaces (De Loof, 1985; Nuccitelli, 1992, 2003a,b; Altizer et al., 2001).

BETSE assumes the existence of two types of electric field (voltage gradient) in the biological tissue (Figure 3B). At the *microscopic* scale (i.e., 10 s of nanometers), very strong voltage gradients are assumed to exist across membranes, gap junctions, and between extracellular spaces (especially across tight junctions) due to the presence of charge at interfaces separated by distances of 10 s of nanometers (Figure 3B). These electric fields, with strength on the order of 0.01–1.0 million volts/meter, are assumed to be the primary drivers of ion flux across membranes and junctions, however, are very short acting due to electrolyte screening. On *mesoscopic* scales (i.e., 10–100 s of micrometers) net ionic current density is assumed to be associated with a longer range, weaker electric field via equation (20) (Figure 3B), which is of much lower strength on the order of 0.2 volts/meter. BETSE assumes the current densities in the environment and in the cell networks are separate.

### 2.6 Standard Equations for Voltage Cross-Check and Validation

#### 2.6.1 Nernst Equation

In cell physiology, two additional equations have been derived from the Nernst–Planck equation for use in *specific* situations involving transport across a membrane: the Nernst equation (Matthews, 2013a) and the Goldman equation (alternatively known as the GHK equation) (Matthews, 2013b).

For the case where the system consists of two compartments separated by a semi-permeable membrane, and the system is at steady-state with both zero ion flux and zero current across the membrane, the Nernst equation (21) can be used to predict the voltage or ratio of concentrations across a membrane:

(21) $$V_{\mathrm{mem}}=\frac{\mathrm{zR}T}{F}\ln\left(\frac{c_{\mathrm{ext}}}{c_{\mathrm{int}}}\right)$$

Note that the Nernst equation (21) should technically only be used for steady-state situations with no flux or current of the ion *c*. A suitable situation would be the equilibrium concentration of a substance such as a reporter dye, which is present at low concentrations and not subjected to active pumping by membrane transporters. BETSE uses the Nernst equation with internally computed intra- and extracellular concentrations of a passively electrodiffusing substance (i.e., modeled reporter dye) to obtain an alternative value for V_mem_, which is used as a cross-check of BETSE-derived concentrations and V_mem_ calculations.

#### 2.6.2 Goldman Equation

The Goldman equation applies for cases where there is a net flux of ions across the membrane, however, the net current is zero, leading to a steady-state or “resting” V_mem_. Due to the action of active ion pumping in living cells, the steady-state V_mem_ represents a dynamic equilibrium with net ion flux but zero current, and can be estimated from the Goldman equation as:

(22) $$V_{\mathrm{mem}}=\frac{RT}{F}\ln\left(\frac{\underset{i}{\sum }P_{{\mathrm{mem}}_{i}}c_{{\mathrm{ext}}_{i}}^{+}+\underset{j}{\sum }P_{{\mathrm{mem}}_{j}}c_{{\mathrm{int}}_{j}}^{-}}{\underset{i}{\sum }P_{{\mathrm{mem}}_{i}}c_{{\mathrm{int}}_{i}}^{+}+\underset{j}{\sum }P_{{\mathrm{mem}}_{j}}c_{{\mathrm{ext}}_{j}}^{-}}\right)$$

In the Goldman equation (22), ions are separated into anions (*c*^−^) and cations (*c*^+^) with concentrations inside (*c_int_*) and outside (*c_ext_*) of the cell membrane. The membrane has a specific permeability *P_mem_* for each unique ion. The Goldman equation is also known as the Goldman–Hodgkin–Katz (GHK) equation.

Note that as the Goldman equation is derived from the Nernst–Planck equation (Matthews, 2013b), the Goldman equation cannot be used to accurately calculate *V_mem_* without developing a circular dependency between concentration and voltage due to an insufficient number of degrees of freedom. Also, the Goldman equation only supplies the transmembrane voltage difference across the membrane, and does not give absolute values for the intra- and extracellular voltages, which are important for calculating cluster-wide bioelectrical signals and states, such as the TEP. However, model parameters computed in BETSE (ion concentrations and membrane permeabilities) were used with the Goldman equation (22) to cross-check and compare final *V_mem_* values obtained using BETSE’s Maxwell Capacitance Matrix voltage solving method defined in section 2.5.

### 2.7 Ion Pumps

Ion pumps were modeled as enzymes using standard Michaelis–Menten enzyme kinetic relations, with reaction rates determined by thermodynamic arguments.

The equilibrium constant of a reaction, *K_eqm_*, can be expressed both in terms of the reaction free energy under standard conditions, $△G_{\mathrm{react}}^{\mathrm{eqm}}$, and in terms of the reaction’s product concentrations (index *k*) and those of its reactants (index *j*) where *a_k_* and *a_j_* represent coefficients of stoichiometry for the reaction (Beard and Qian, 2007; Pekar, 2015):

(23) $$K_{\mathrm{eqm}}=\exp\left(-\frac{△G_{\mathrm{react}}^{\mathrm{eqm}}}{RT}\right)=\frac{\sum c_{k}^{a_{k}}}{\sum c_{j}^{a_{j}}}$$

The electrochemical potential of a substance at concentration *c_i_* with charge *z_i_* in a region where there is a voltage *V* is expressed:

(24) $$\mu_{i}=\mu_{o}+RT\ln\left(c_{i}\right)+z_{i}FV$$

Furthermore, the overall free-energy of a reaction is described as the sum of the (electro)chemical potentials of its products (index *k*) minus those of its reactants (index *j*) where *a_k_* and *a_j_* represent coefficients of stoichiometry for the reaction:

(25) $$\Delta G_{\mathrm{reaction}}=\sum a_{k}\mu_{k}-\sum a_{j}\mu_{j}$$

Using the Na/K-ATPase pump as an example, the overall reaction for the Na/K-ATPase pump is:

(26) $$3c_{\mathrm{Na}}^{\mathrm{in}}+2c_{K}^{\mathrm{out}}+\mathrm{ATP}↔3c_{\mathrm{Na}}^{\mathrm{out}}+2c_{K}^{\mathrm{in}}+\mathrm{ADP}+P$$

From the abovementioned fundamental chemical principals, the overall free energy, Δ*G_pump_*, for the Na/K-ATPase pump reaction can be expressed (Smith and Crampin, 2004):

(27) $$\begin{array}{l} \begin{array}{rl} \Delta G_{\mathrm{pump}} & =△G_{\mathrm{ATP}}^{o}+RT\ln\left(\Omega \right)-FV_{\mathrm{mem}}\\ \Omega & =\frac{\mathrm{cADP}\mathrm{cP}\mathrm{cN}a_{\mathrm{out}}^{3}cK_{\mathrm{in}}^{2}}{\mathrm{cATP}\mathrm{cN}a_{\mathrm{in}}^{3}cK_{\mathrm{out}}^{2}} \end{array} \end{array}$$

when Δ*G_pump_* = 0, the reaction is at equilibrium. Using equation (23), an expression for the Na/K-ATPase pump reaction equilibrium constant in terms of the standard free energy for ATP hydrolysis and cell *V_mem_* is:

(28) $$K_{\mathrm{NaKATP}}^{\mathrm{eqm}}=\exp\left(\frac{-\Delta G_{\mathrm{ATP}}^{o}+FV_{\mathrm{mem}}}{RT}\right)$$

Following with basic Michaelis–Menten enzyme kinetics, an estimate for the rate of the reversible enzymatic pump reaction follows as:

(29) $$\begin{array}{llll} \alpha_{\mathrm{NaKATP}} & =\alpha_{o}\left(\frac{\frac{\mathrm{cATP}}{K_{\mathrm{ATP}}}\frac{{\mathrm{cNa}}_{\mathrm{in}}}{K_{\mathrm{Na}}}\frac{cK_{\mathrm{out}}}{K_{K}}}{\left(1+{\mathrm{cATPK}}_{\mathrm{ATP}}\right)\left(1+{\mathrm{cNa}}_{\mathrm{in}}K_{\mathrm{Na}}\right)\left(1+cK_{\mathrm{out}}K_{K}\right)}\right)\; & & \;\\ & \;\times \left(1-\frac{\Omega }{K_{\mathrm{NaKATP}}^{\mathrm{eqm}}}\right) \end{array}$$

Values for the Michaelis constants *K_Na_* = 5.0, *K_K_* = 0.2, and *K_ATP_* = 0.15 were obtained from references (Munzer et al., 1994; Vrbjar et al., 1994). Values of *α_o_* were roughly calibrated to Na/K-ATPase pump rates reported for *Xenopus* oocytes (Costa et al., 1989).

In addition to Na/K-ATPase pumps, BETSE can optionally simulate Ca-ATPase, H/K-ATPase, and V-ATPase pumps using free-energy regulated pumping rates analogous to that outlined above for the Na/K-ATPase pump.

### 2.8 Voltage-Gated Channels

A range of voltage-gated channel types have been implemented in BETSE using Hodgkin–Huxley style differential equations to define the state of membrane diffusion to a specific ion (e.g., Na^+^) as a function of V_mem_ and time. Specific parameters and functional relations were obtained from the online database, *Channelpedia* (Ranjan et al., 2011).

The present work specifically uses a combined generic voltage-gated sodium channel (NaV) from (Hamill et al., 1991), and a delayed-rectifier voltage-gated potassium channel (KV1.2) from (Sprunger et al., 1996), to generate excitable signals. A standard Hodgkin–Huxley style model uses an electrical equivalent circuit equation to determine changes to current and voltage across a membrane, with a set of differential equations controlling the conductance of the membrane (Nelson, 2004). Since conductance is proportional to the membrane diffusion constant for a particular ion [see equations (12) and (13)], BETSE uses the same Hodgkin–Huxley style equations developed to describe membrane conductivity state to describe the membrane diffusion state of a particular ion, updating subsequent changes to currents and voltages using its own methods, as described in the above. Details regarding voltage-gated channel dynamics are specified in Supplementary Material.

### 2.9 Gap Junctions

Gap junctions were modeled as (optionally) voltage-sensitive conduits influencing the intercellular diffusion coefficient for all ions uniformly via a diffusion–constant scaling factor, $\beta_{\mathrm{GJ}}^{o}$. Simulated transport through GJ used the Nernst–Planck equation [equation (1)] to update concentration of all ions moving under intercellular concentration and voltage gradients. In the absence of GJ, cells were modeled to have an intercellular diffusion coefficient of zero ($\beta_{\mathrm{GJ}}^{o}=0$). Medium-high GJ connectivity corresponded to $\beta_{\mathrm{GJ}}^{o}$ = 1.0 × 10^−6^, an intercellular diffusion coefficient of approximately 1.0 × 10^−15^m^2^/s. Assuming 1.0 × 10^5^ GJ per cell, and cylindrical GJ with pore diameter of 1.5 nm and length of 26 nm, this corresponds to individual GJ conductance of 68 pS, which is in the mid-range of reported GJ conductances (Goodenough and Paul, 2009).

Voltage gating of GJ was described using the kinetic model of (Harris et al., 1983), which calculates GJ open/closed state (*β_GJ_*) dependence on voltage difference across the gap junction (*V_GJ_*) and time. Specific details regarding voltage gating of GJ are described in Supplementary Material.

### 2.10 Tight Junctions

Multicellular organs and organisms develop very low-permeability TJ at their exterior boundary, which are involved in creating the important TEP voltage gradient across the organ/organism boundary. In BETSE, the degree of movement of ions in both chemical and electrical gradients was handled by considering three interconnected, but distinct transport pathways (transmembrane, intercellular, extracellular), with the possibility for spatially varying diffusion coefficients within extracellular regions, with low diffusion at the boundary simulating the presence of TJ (see Figure 3).

### 2.11 Electroosmosis

Electroosmotic flows are a hypothesized transport mechanism in biological systems (Andreev, 2013). BETSE assumed that electroosmosis may occur through small channel structures of the heterogeneous tissue, such as gap junctions between cells (gap junction radius *r_gj_* ~ 5 to 8 nm) and the narrow channels (*d_ecm_* ~10 to 30 nm) formed by extracellular spaces.

Our simple estimate used a modified version of the Hagen–Poiseuille equation (Gao et al., 2011) to estimate electroosmotic fluid flows between the small channels represented by gap junction connected cells or extracellular spaces:

(30) $${\vec{u}}_{o}=\frac{\pi r^{4}}{8\mu }{\vec{F}}_{e}$$

where ${\hat{F}}_{e}$ is a volume force generated by electrostatic forces resulting from a voltage gradient (electric field $\vec{E}$) between two cells or extracellular spaces:

(31) $${\vec{F}}_{e}=\rho_{e}\vec{E}$$

As mass cannot be created or destroyed, fluid flow velocity must be a divergence-free field, which physically corresponds to the development of internal pressures resisting fluid flow. The internal pressure was estimated as:

(32) $$P_{\mathrm{int}}=\nabla^{-2}\left(\nabla \cdot {\vec{u}}_{o}\right)$$

The gradient of the internal pressure was used to correct the velocity calculated from equation (30), yielding the final estimate for electroosmotic fluid velocity:

(33) $$\vec{u}={\vec{u}}_{o}-\nabla P_{\mathrm{int}}$$

Electroosmotic fluid velocities were treated separately in the intra- and extracellular spaces.

### 2.12 Other Biophysical Phenomena

Details regarding the implementation of other biophysical phenomena, such as lateral self-electrophoretic/electroosmotic transport of ion pumps and channels in cell membranes, and the development of osmotic and hydrostatic pressures, are discussed in Supplementary Material.

## 3 Results

### 3.1 Model Validation and Resting V_mem_ Regulation in Isolated Cells

Simulations 1, 2, and 3 were used to validate the core BETSE model by determining its ability to predict resting V_mem_ and expected V_mem_ dynamics under a series of perturbations for isolated cells not connected by TJ or GJ (single-cell behavior). Validations also checked that equilibrium concentration profiles of an electrodiffusing charged molecule (simulated reporter dye) showed values predicted from the Nernst equation (21). The behavior of voltage-gated channels was explored in simulation 4.

#### 3.1.1 Simulation 1: Prediction of *Xenopus* oocyte V_mem_

The first validation step used experimentally derived input values (membrane permeability and environmental ion concentrations), comparing simulated output to experimentally observed parameters (V_mem_).

Experimentally observed membrane ion permeabilities and extracellular ion concentrations of Na^+^, K^+^ and Cl^−^ obtained from *Xenopus* oocytes (Costa et al., 1989), were used as input parameters (Table 2). The simulation was performed on a small network of 35 isolated cells for 30 min of simulated time. The resulting BETSE-derived V_mem_ and intracellular ion concentrations were compared to those observed experimentally for *Xenopus* oocytes with the same membrane ion permeabilities and under the same extracellular ion concentrations (Costa et al., 1989). After 30 min of simulation, steady-state V_mem_ and intracellular ion concentrations calculated by BETSE showed <10% difference between experimentally determined values measured from *Xenopus* oocytes (Table 2).

**Table 2:** **Input membrane permeabilities (P_mem_i_) and simulated V_mem_, and ion concentrations in the extra- and intracellular spaces at steady-state (30 simulated minutes) for the resting membrane ion permeability profiles of *Xenopus* oocytes bathed in Ringer’s solution, as reported elsewhere (Costa et al., 1989)**.

Variable	BETSE (extracellular)	Experimental (extracellular)	% Difference
P_mem_Na_ [nm/s]	0.537	0.537	/
P_mem_K_ [nm/s]	1.765	1.765	/
P_mem_Cl_ [nm/s]	0.138	0.138	/
Na^+^ [mmol/L]	9.21 (115.0)	10.1 (114.0)	8.8 (0.9)
K^+^[mmol/L]	100.7 (2.5)	109.5 (2.4)	8.0 (4.2)
Cl^−^[mmol/L]	39.95 (111.5)	37.7 (116.4)	6.0 (4.2)
Ca^2+^[nmol/L]	104.0 (2.0 × 10^6^)	N/A	N/A
X^−^[mmol/L]	50.0 (10.0)	N/A	N/A
pH	7.3 (7.6)	7.3 (7.5)	0.0 (1.3)
V_mem_ [mV]	−37.6	−39.1	3.8

*For ion concentration columns, intracellular concentrations are stated with extracellular concentrations in parentheses. Concentrations of charged proteins (and other negatively charged macromolecules) are indicated by “X^−^.” Steady-state V_mem_ and ion concentrations calculated by BETSE show <10% difference between experimentally determined values measured from *Xenopus* oocytes (Costa et al., 1989)*.

#### 3.1.2 Simulation 2: Resting V_mem_ as an Attractor State

**Figure 5:**
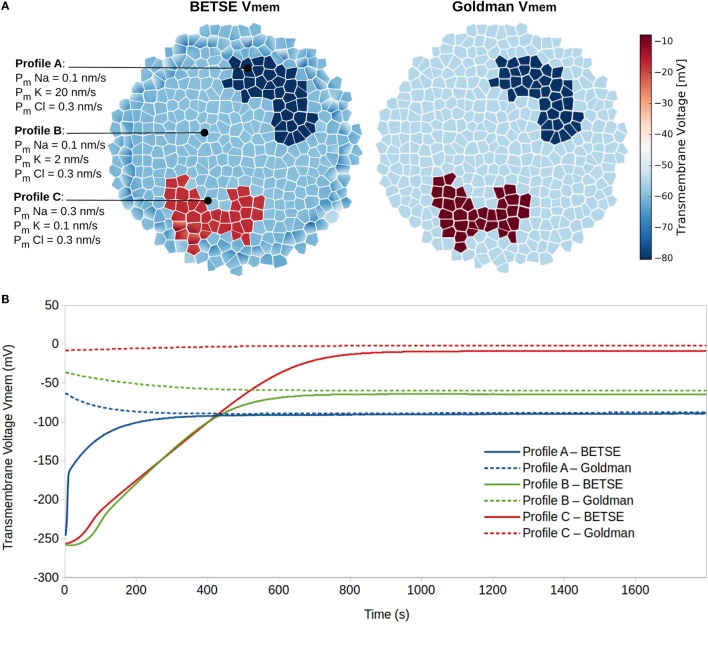
**Resting potentials (steady-state V_mem_) are states of dynamic equilibrium highly influenced by cell membrane ion permeability profiles**. Three different cell membrane ion permeability profiles are defined for cells in a cluster **(A)**. Starting from equal concentrations of ions in the intra- and extracellular environments (see Table 3), BETSE reaches a steady-state V_mem_ value closely predicted by the Goldman equation for the three cell profile types **(A,B)**.

Simulation 2 explored resting V_mem_ as a dynamic systems attractor state, reaching a characteristic value even with highly divergent initial conditions. This is an important property to understand, in light of the significant robustness of biological pattern regulation. The simulation was performed on a small cluster of 183 isolated cells, which were not connected by gap or tight junctions, where cells in different regions were assigned to one of three membrane ion permeability profiles (Figure 5A). The membranes of profile A, B, and C cells had high, medium, and low sustained K^+^ membrane permeability, respectively (Figure 5). All other parameters associated with cells in the three profiles were identical.

The simulation began with a non-physiological starting state featuring equal concentrations of ions in both the intra- and extracellular environments (starting concentrations are those typical of human plasma and are given in Table 3) and with no voltage in any part of the system (V_mem_ = 0 in all cells).

**Table 3:** **Simulated ion concentrations in the extracellular and intracellular spaces at time zero, and at steady-state (20 simulated minutes) for three different membrane ion permeability profiles defined in Figure 5**.

Variable	Extra	Intra (0 s)	Intra A (20 min)	Intra B (20 min)	Intra C (20 min)
Na^+^ [mmol/L]	145.0	145.0	7.4	5.38	3.18
K^+^ [mmol/L]	5.0	5.0	143.3	151.1	177.2
Cl^−^[mmol/L]	105.0	105.0	30.8	34.7	52.5
Ca^++^ [nmol/L]	1.0 × 10^6^	1.0 × 10^6^	145.6	79.5	15.4
X^−^[mmol/L]	10.0	80.0	80.0	80.0	80.0
pH	7.40	7.40	7.62	7.64	7.70
Dye^−^[*μ*mol/L]	1.0	1.0	0.043	0.112	0.822
V_mem_ Dye [mV]	/	0.0	−84.1	−58.5	−5.2
V_mem_ Goldman [mV]	/	0.0	−82.3	−54.1	+ 1.1
V_mem_ BETSE [mV]			−83.9	−58.5	−5.2

*The system began (*t* = 0 s) with equal intra- and extracellular concentrations and V_mem_ = 0 (zero net charge in cell or environment). Extracellular concentrations remained fixed throughout the simulation. Concentration of charged proteins and other negatively charged macromolecules are indicated by “X^−^.” An electrodiffusing negatively charged reporter dye was also included in the simulation (“Dye^−^”) and assumed to be at low concentrations and, therefore, not influencing V_mem_. The Nernst equation was used with 20-min BETSE-simulated intra- and extracellular dye concentrations as an alternative V_mem_ estimate (“V_mem_ Dye”). Steady-state V_mem_ calculated by BETSE or using the GHK equation are also listed*.

The Goldman equation [equation (22)] was used with membrane permeability and ion concentration values available at each time step to predict V_mem_ using conventional measures and provide an indicator of expected resting V_mem_ for each of the three profiles.

The simulation shows that after 20 simulated minutes, the BETSE-calculated V_mem_ closely approaches (<10% discrepancy) the value predicted by the Goldman equation [equation (22)] for the three cell membrane-permeability profile types (Figure 5). As is expected from theory (Matthews, 2013b), the steady-state V_mem_ value complying with the Goldman equation is reached when the net trans-membrane current reaches zero (data not shown).

In addition to the six major ions, an electrodiffusing negatively charged “reporter dye” was also included in the simulation (“Dye^−^,” Table 3) and assumed to be at low concentrations and, therefore, not influencing V_mem_. The Nernst equation [equation (21)] was used with BETSE-simulated intra- and extracellular dye concentrations as an alternative V_mem_ estimate (“V_mem_ Dye,” Table 3); results are virtually identical between the direct-BETSE and dye-estimated V_mem_ values.

Notably, while concentrations in intra and extracellular spaces began equal, at steady-state (20 simulated minutes) intracellular ion concentrations were within expected physiological ranges (Veech et al., 1995; Lodish et al., 2000; Wright, 2004) (Table 3).

In addition to model validation, this simulation emphasizes resting V_mem_ of isolated cells as stable states of dynamic equilibrium that are attractor states with final values highly influenced by cell membrane ion permeability profiles. As expected, increased membrane permeability to K^+^ (simulating increased expression of K^+^ leak channels) leads to higher degrees of V_mem_ hyperpolarization (Lodish et al., 2000; Wright, 2004).

#### 3.1.3 Simulation 3: Influential Factors and Perturbation of Resting V_mem_

**Figure 6:**
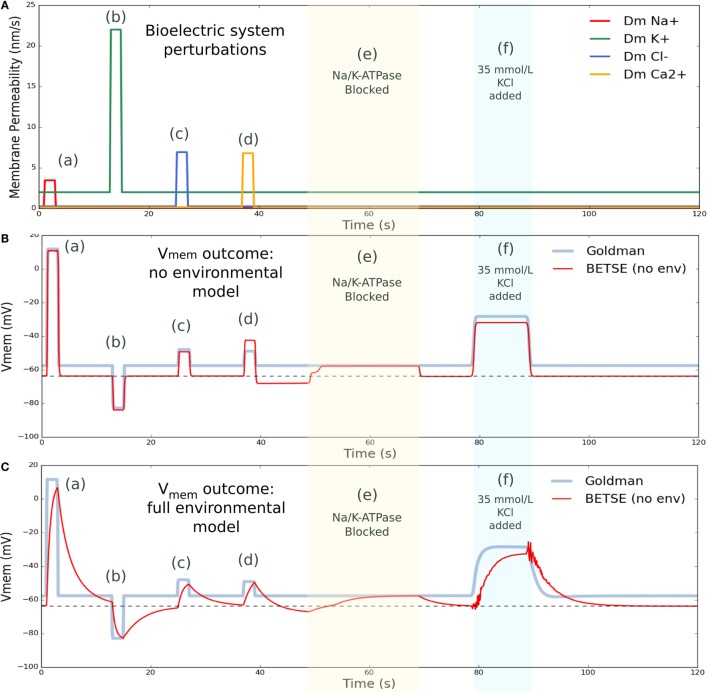
**V_mem_ changes with perturbation to membrane permeability or external ion concentrations**. **(A)** shows membrane permeability to specific ions, which was altered via various forced changes during the simulation. Intervention (a) increased membrane permeability to Na^+^ by a factor of 25 from 1 to 3 s. Intervention (b) increased permeability to K^+^ by a factor of 10 from 13 to 15 s. Intervention (c) increased permeability to Cl^−^ by a factor of 25 from 25 to 27 s, and intervention (d) increased permeability to Ca^2+^ by 50 from 37 to 39 s. In between membrane permeability perturbations, permeability returned to original values. Intervention (e) blocked activity of the Na/K-ATPase pump from 49 to 69 s. Intervention (f ) temporarily increased the extracellular concentration of K^+^ by introducing 35 mmol/L KCl at the global boundaries from 79 to 89 s, returning to 5 mmol/L at the boundary after 26 s. **(B)** shows BESTE-calculated V_mem_ in comparison to Goldman-derived V_mem_ for a single cell undergoing the applied interventions, for the situation where instantaneous mixing was assumed in the environmental space (i.e., “cytosol only” simulation). Dashed black line in **(B)** indicates the value for resting V_mem_ (−63.7 mV). **(C)** shows BETSE-calculated V_mem_ in comparison to Goldman-derived V_mem_ for a single cell undergoing the same various applied interventions, for the situation where individual extracellular spaces and environmental electrodiffusion were modeled (i.e., “environment modeling” simulation). Dashed black line in **(C)** indicates the value for resting V_mem_ (−63.7 mV).

Simulation 3 explored factors influencing resting V_mem_ in isolated cells, and also demonstrated the ability for cell V_mem_ to return to its resting value after a perturbation (Figure 6). As factors, such as membrane permeability to specific ions, ion pump rates, and the influence of environmental ion concentrations, such as high extracellular K^+^ levels, are well known to affect individual cell V_mem_ (Lodish et al., 2000; Wright, 2004), this simulation (Figure 6) is also a model validation. The simulation was performed on the same cluster used in Simulation 2 (see Figure 5A), with membrane manipulations applied to, and V_mem_ monitored in, a profile B cell of the cluster. Initial conditions for Simulation 3 were those of the *final* Simulation 2, with extra/intracellular ion concentrations and V_mem_, as listed for the 20 min time point in Table 3.

The Goldman equation [equation (22)] was used with membrane permeability and ion concentration values available at each time step to predict V_mem_ using conventional measures and provide an indicator of expected V_mem_.

“Cytosol only” and “environment modeling” are two simulation modes available in BETSE. In “cytosol only” mode, a simple simulation is performed, which assumes instantaneous mixing of fluxes into the environment, where extracellular spaces and free diffusion in the environment are not modeled, and V_mem_ is calculated assuming the cell is a simple capacitor via the charge inside the cell and the relation $V_{\mathrm{mem}}=\frac{1}{\mathrm{Cm}}Q_{\mathrm{cell}}$. The “environment modeling” mode calculates a full extracellular environment using the Maxwell Capacitance Matrix method to solve for voltages, as outlined in the Methods section. The V_mem_ data presented in Figure 6B is from the “cytosol only” simulation mode, while that from 6C is from the “environment modeling” simulation mode. These two types of simulation modes were compared to illustrate the effect of including extracellular matrix and environmental transport in the bioelectrical model.

Various membrane permeability manipulations, in addition to a block of the Na/K-ATPase pump, and an increase in extracellular K^+^ levels, were simulated (Figure 6). Membrane permeability manipulations effectively simulate the transient opening of an ion channel. The first intervention temporarily increased (from 1 to 3 s) the cell’s membrane permeability to Na^+^ by a factor of 25, leading to a characteristic, pronounced depolarization. The next intervention temporarily increased (from 13 to 15 s) the cell’s membrane permeability to K^+^ by a factor of 10, and generated a characteristic hyperpolarization of V_mem_. Subsequent interventions increased the cell’s membrane permeability to Cl^−^ by a factor of 25 from 25 to 27 s, creating an expected depolarization, and increased the membrane permeability to Ca^2+^ by 50 from 37 to 39 s with the expected depolarization effect. The Na/K-ATPase pump activity was blocked from 49 to 69 s, during which time the V_mem_ for both simulation modes converged at precisely the Goldman V_mem_ prediction (Figure 6). This result is expected as the Na/K-ATPase pump activity generates an electrogenic current that is not considered in the Goldman analysis (the Goldman equation requires zero net current across the membrane, as discussed previously). Finally, extracellular K^+^ concentration was increased by introducing 35 mmol/L KCl at the global boundaries from 79 to 89 s, which returned to 5 mmol/L at the boundary after the perturbation interval, and resulted in a characteristic, well-known V_mem_ depolarization (Delamere and Duncan, 1977).

As can be seen in Figure 6, cell V_mem_ naturally returns to its resting value of −63.7 mV after each perturbation is complete. While overall, V_mem_ responses for both the “cytosol only” and “environmental modeling” simulation modes captured all main features predicted by the Goldman equation, the “environmental modeling” mode, which includes simulation of extracellular spaces and transport in the environment, showed slower and more complex responses than the “cytosol only” mode.

These results illustrate how physiological circuits implement stability with respect to bioelectric state, as, for example, observed in applications of optogenetics to developing systems (Adams et al., 2013).

#### 3.1.4 Simulation 4: Resting V_mem_ and Cell Excitability

**Figure 7:**
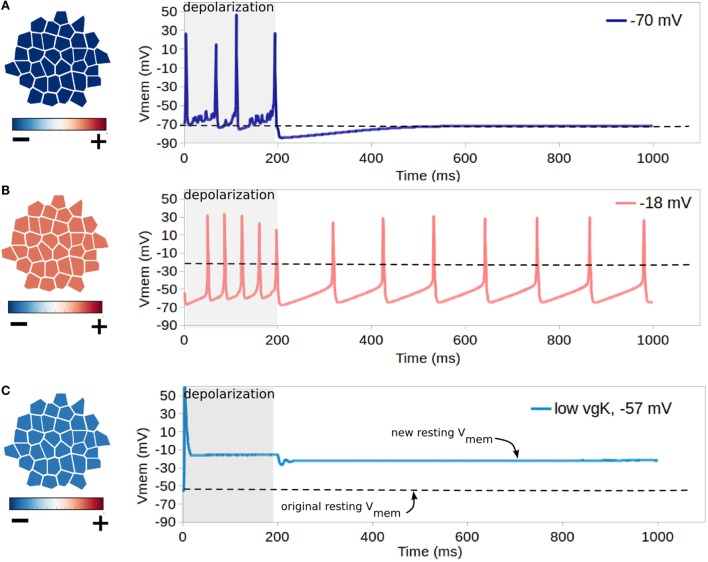
**Influence of resting V_mem_ on cell excitability**. Each simulation shown in rows **(A–C)** features cells with different resting V_mem_ (as indicated in leftmost panels) which is induced by altering cell levels of K^+^ leak channels (altering non-dynamic membrane permeability to K^+^). In each simulation, all cells have identical NaV and KV1.2 channels, and a forced depolarization is applied to one randomly selected cell of the cluster from a time of 1–200 ms. For cells with the lowest resting potential of −70 mV [row **(A)**], the forced depolarization leads to the firing of four action-potential-like signals, with excitable activity ceasing with the forced depolarization after 200 ms. For the cluster with the low resting potential of −18 mV [row **(B)**], the forced depolarization leads to a periodic self-excitation with a period of about 100 ms, which lasts long after the forced depolarization ceases. **(C)** shows that combined activity of dynamic channels with variable expression levels can generate resting V_mem_ bi-stability, as for cells with an original resting potential of −57 mV, expression of NaV channels with low expression of KV1.2 transitions the system into a depolarized V_mem_ of approximately −14 mV after a single forced depolarization.

Simulation set 4 validated the expected function of voltage-gated Na^+^ and K^+^ channels, highlighted the ability for resting V_mem_ to control cell excitability, and examined the possibility of low voltage-gated K + expression in relation to voltage-gated Na + expression to effect resting V_mem_. Simulations were performed on a cluster of 35 cells, which were connected by non-voltage sensitive GJ, and were without TJ. An initialization simulation without voltage-gated channels was run on each cluster to bring cells to equilibrium resting state. Each simulation shown in rows A–C of Figure 7 features cells with different resting V_mem_, which is accomplished by altering levels of K^+^ leak channels (altering non-dynamic membrane permeability to K^+^). In simulations A and B of Figure 7, all cells have identical expressions of NaV and KV1.2 channels, with a net maximum membrane permeability of 2667 nm/s and 667 nm/s for NaV and KV1.2 channels, respectively. The resting V_mem_ of cells in simulation A was −70 mV, while those of simulation B weremuch higher at −18 mV. Simulation C of Figure 7, studied cells with a resting potential of −57 mV and expressions of NaV and KV1.2 channels (homogeneous expressions in the cell population), with a net maximum membrane permeability of 2667 nm/s and 67 nm/s for NaV and KV1.2 channels, respectively. This simulated a deficiency of voltage-gated potassium channels – a phenotype occurring in certain metastatic cancers (Djamgoz, 2014). For each simulation, a forced depolarization is applied to one randomly selected cell of the cluster from a time of 1–200 ms to induce excitable activity.

For cells with the lowest resting potential of −70 mV (Figure 7A), the forced depolarization leads to the firing of four action-potential-like signals, with excitable activity ceasing with the forced depolarization after 200 ms. However, for the cluster with the low resting potential of −18 mV (Figure 7B), the forced depolarization leads to a periodic self-excitation with a period of about 100 ms, which lasts long after the forced depolarization ceases. This demonstrates the well-known expected behavior of cells with resting V_mem_ higher than the activation threshold of NaV, such as the pacemaker cells of the heart, to enter periodic self-excitations for an indefinite period of time (Roberts and Stirling, 1971; Onganer et al., 2005; Matthews, 2013a). For hyperpolarized cells with resting V_mem_ of −57 mV with expression of NaV and simulated deficiency of voltage-gated potassium channels (Figure 7B), our simulations indicate that the forced oscillation creates a single action potential, with the resting potential being altered in the long term to a much more depolarized value of −14 mV. These simulations demonstrate both the importance of resting potential in controlling cell excitability, with more depolarized cells showing capacity for self-excitation (Figures 7A,B), and also the capacity for irregular expression of excitable channels to potentially alter the resting V_mem_ (Figure 7C), which may assist in explaining the sustained depolarization of some cancer cells (Djamgoz, 2014).

### 3.2 Impacts of Multicellular V_mem_ Gradients

#### 3.2.1 Simulation 5: Effect of Heterogeneous V_mem_ on Physiological Properties

**Figure 8:**
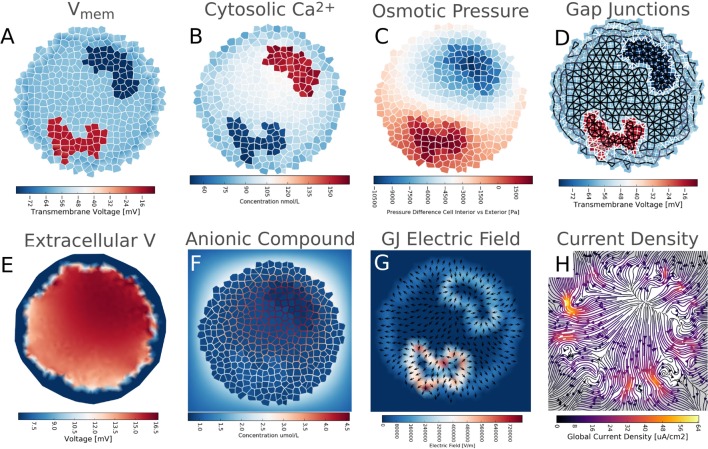
**Differences in *V_mem_* between different cells in a cluster have various biophysical influences on the cluster as a whole**. **(A)** *V_mem_* pattern featuring a depolarized spot of cells in the lower left side and a hyperpolarized spot of cells in the upper right side of a circular cell cluster **(A)** results in differences in cytosolic Ca^2+^ levels **(B)**, osmotic pressure **(C)**, patterns of voltage-sensitive gap junction conductivity [**(D)**, where black is open and white is closed], and small interior environmental voltage gradients **(E)**. Patterns of *V_mem_* also influence the cytosolic and extracellular concentration of a negatively charged anionic compound **(F)**, induce strong nano-scale electric fields between gap junctions **(G)**, and generate a characteristic long-range pattern of total ionic current density and macroscopic electric field **(H)**.

Simulation 5 investigated the physiological impacts of a heterogeneous V_mem_ pattern in a cellular collective. A cluster of 794 cells with a diameter of 375 *μ*m, boundary TJ with a diffusion scaling of 1.0 × 10^−5^, and GJ connectivity with a value of $\beta_{\mathrm{GJ}}^{o}$ = 1 × 10^−7^was utilized. Initial values for concentrations and voltages in the simulations were those of the final simulation for profile B cells, Table 3. Membrane permeability of cells varied over space in the same pattern and using the same three profiles defined in Figure 5A. The result was a stable pattern of resting V_mem_ featuring a depolarized spot of cells in the lower left side (V_mem_ ~ −20 mV) and a hyperpolarized spot of cells in the upper right side of a circular cell cluster (V_mem_ ~ −60 mV). Each region was surrounded by cells with a mean V_mem_ of approximately −45 mV (Figure 8A).

The presence of regional V_mem_ differences was found to have various influences on the cluster as a whole. Heterogeneous V_mem_ induced differences in cytosolic Ca^2+^ levels (Figure 8B) in a manner inversely proportional to cell V_mem_, with the most hyperpolarized cells having cytosolic Ca^2+^ of over 150 nmol/L while the most depolarized contained <60 nmol/L. By contrast, a hypothetical negatively charged anionic signaling molecule develops a *cytosolic* concentration profile in direct correspondence to V_mem_ values (inverse to that of cationic Ca^2+^), but due to the presence of TJ, which enable the formation of extracellular voltages due to charge internalization (Figure 8F), the anionic substance concentrates in extracellular spaces around *hyperpolarized* cells (Figure 8F).

Heterogeneous V_mem_ was also seen to produce significant osmotic pressure differences between cells of different resting potential, with more hyperpolarized V_mem_ leading to lower osmotic pressure than more depolarized V_mem_ (Figure 8C). This is consistent with expectations, as in simulation 5 more hyperpolarized cells have higher K^+^ leak channels, which means more K^+^ is moving out of the cell and into extracellular spaces with expected water movement from the cytosol to the extracellular space to compensate for movement of salt (i.e., lowering of osmotic pressure). By contrast, depolarized cells of the simulation have higher levels of Na^+^ leak permeability, which means more Na^+^ is moving from the extracellular space to the cytosol with expected water movement from the extracellular space to the cell to compensate (increase of intracellular osmotic pressure). Depending on the mechanical properties of cells, these osmotic pressures may translate into cell volume changes and hydrostatic pressures and pressure gradients (body forces).

Voltage-sensitive Gap junctions connecting cells responded to voltage gradients created by V_mem_, closing to minimum conductivity values and isolating the two regions of differential V_mem_ from the remainder of the cluster (Figure 8D). The V_mem_ pattern in this example generated electric fields of up to ~6.5 × 10^5^ V/m acting (over short spatial distances of 26 nm) between interfacing cell membranes of GJ networked cells (Figure 8G).

Heterogeneous V_mem_ in a GJ networked multicellular cluster also was found to induce a long-range pattern of total ionic current density up to 60 *μ*A/cm^2^ in magnitude (Figure 8H).

We conclude that stable patterns of resting V_mem_ have numerous, significant impacts on the cluster as a whole, altering concentration profiles of key signaling moieties, inducing physiological pressures and forces, and establishing long-range patterns of ion transport and macroscopic electric field.

### 3.3 Factors Influencing Resting V_mem_ in Networked Cells

#### 3.3.1 Simulation 6: Gap Junction Connectivity

**Figure 9:**
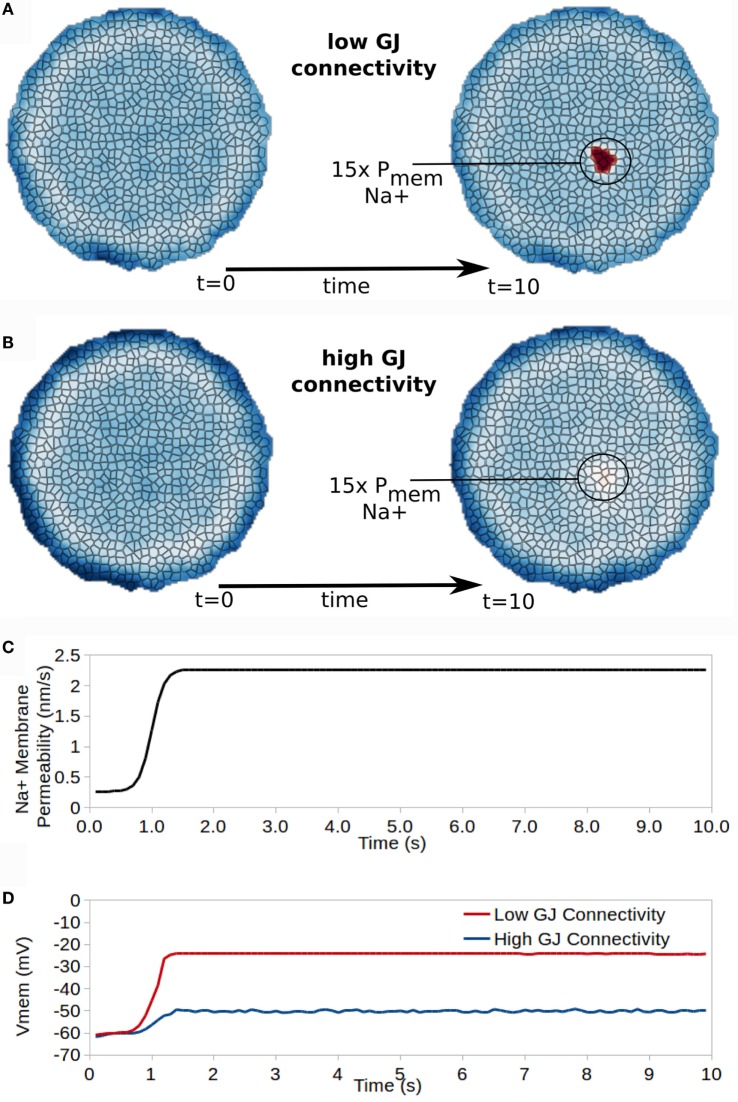
**Gap junction connectivity has a strong affect on the ability for cells with altered properties to manifest different bioelectrical states in the collective**. A small patch of cells developing 15× increased Na^+^ permeability transitions those cells into a new resting V_mem_ for cells with low GJ connectivity **(A)** but not for cells with high GJ connectivity **(B)**. **(C)** shows the time dependence of Na membrane permeability for the affected patch of cells, while **(D)** shows the time course of V_mem_ for a cell in the affected patch of cells for high and low GJ connectivity clusters.

To understand group dynamics and the dynamics of bioelectric states in an electrically coupled tissue, this simulation explored the influence of GJ connectivity on cell resting V_mem_ for a small group of cells (encircled in Figure 9) with 15× increased Na^+^ membrane permeability (simulating an increased expression of an open Na^+^ ion channel). The multicellular cluster contained 794 cells, and had a diameter of 375 *μ*m. Cells were connected by GJ at interfacing membranes. The simulation began with values for intra/extracellular concentrations and V_mem_ obtained after a 20 minute initialization simulation, which were similar to those listed in Table 3’s 20 min time point for profile B cells. All cells began with identical membrane permeability profiles with values of profile B cells as listed in Figure 5A.

Cells in a first simulation (low GJ connectivity) had an intercellular (GJ mediated) free-diffusion scaling of $\beta_{\mathrm{GJ}}^{o}$ = 1.0 × 10^−7^. For a cell with 1.0 × 10^5^ GJ in total, this corresponds to an individual GJ conductivity of approximately 68 pS.

Cells in a second simulation (low GJ connectivity) had an intercellular (GJ mediated) diffusion scaling constant of $\beta_{\mathrm{GJ}}^{o}$ = 1.0 × 10^−6^. For a cell with 1.0 × 10^5^ GJ in total, this corresponds to an individual GJ conductivity of approximately 6.8 pS.

For both high and low GJ connectivity simulations, at t = 1.0 s of the simulation Na^+^ membrane permeability of a small patch of cells (circled in Figures 9A,B) was increased by 15× and remained increased for the duration of the simulation (Figure 9C). This simulates the increased expression, or opening, of a Na^+^ ion channel in this small patch of cells, but not in the remaining cells of the cluster.

Effects on V_mem_ vary significantly between cells with high or low GJ connectivity (Figure 9). For a cluster with low GJ connectivity, the 15× increase in Na^+^ permeability leads to approximately 40 mV depolarization of V_mem_, which remains stable as a new resting V_mem_ state divergent from that of surrounding cells (Figure 9D). However, the cluster with high GJ connectivity shows only 10 mV depolarization in V_mem_ with the 15× increase in Na^+^ permeability (Figure 9D).

We conclude that the characteristics of GJ connectivity in a cluster have a significant influence in specifying resting V_mem_ for cells with heterogeneous ion channel characteristics, and may, therefore, be expected to play important roles in morphogenesis and the development of cancer, which both require the development of differential V_mem_ states from a homogeneous collective.

#### 3.3.2 Simulation 7: Tight Junction Connectivity

**Figure 10:**
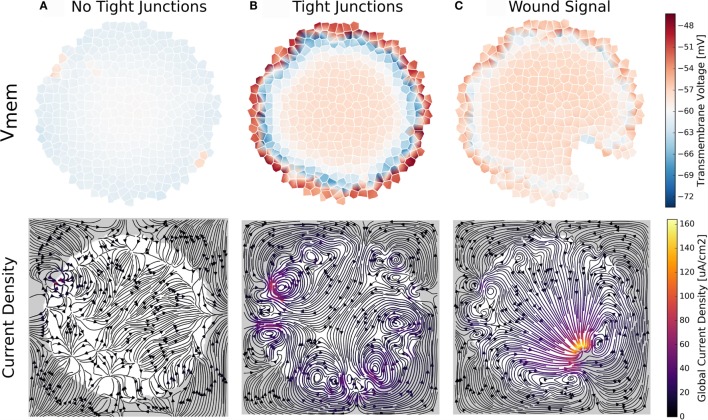
**A trans-boundary voltage difference (trans-epithelial potential; TEP) appears across the outer boundary of cell clusters when ion transport between extracellular spaces at the cluster boundary is limited by simulated tight junctions (B,C)**. This trans-boundary voltage does not occur when ion transport in extracellular spaces is similar to free-diffusion values for ions **(A)**. With TJ present, wounding leads to a characteristic bioelectrical cluster state **(C)** where ion current flows out of the wounded area, returning to the cluster at both sides of the wound, and establishing a long-range (100 s of micrometers) current flow and macroscopic electric field in the cluster **(C)**.

**Figure 11:**
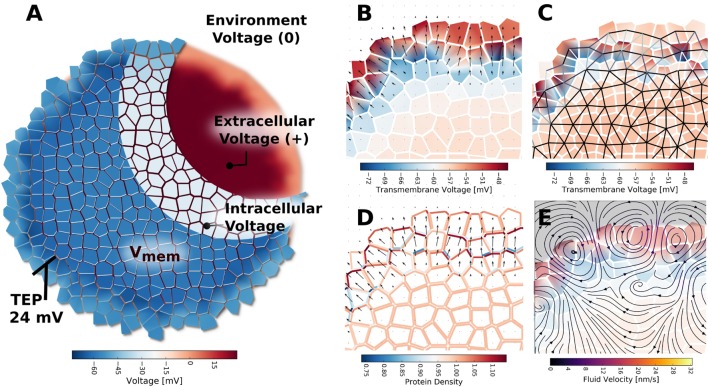
**The TEP appearing across the outer boundary of cell clusters, when ion transport between extracellular spaces at the cluster boundary is limited by simulated tight junctions, was indicated to form because of charge and voltage build up in the extracellular environment (A)**. The TEP strongly polarizes individual cells in the outer layer of the cluster **(B)**, and results in an electric field at the cluster boundary. The strong voltage gradients of the TEP were predicted to create spatial patterns of GJ voltage gating, whereby cells in the outer layer and cluster interior form two networked groups, but cells between the outer layer and cluster interior are separated by closed and minimally conductive GJ [**(C)**, black represents fully open GJ, white represents fully closed GJ]. Patterns of global ionic current density (not shown) and electroosmotic fluid flow **(E)** are strongest in the cluster across the TEP. The strong electric field and electroosmotic flows at the cluster boundary are predicated to result in lateral self-electrophoresis/electroosmosis of ion pumps and channels in the membranes **(D)**, which further alters current, flow, TEP, and *V_mem_* patterns of the cluster.

In this set of simulations, a circular cluster with limited diffusion at the outer environmental cluster boundary was simulated to explore the effect of tight junctions (Figures 10 and 11). Initial values for concentrations and voltages in the simulations were those of the final simulation for profile B cells, Table 3. Simulations investigated (1) the general effect of TJ presence, which decreased extracellular boundary diffusion constants by 1.0 × 10^−5^ from free diffusion values (Figures 10B and 11); (2) the effect of no TJ by leaving extracellular boundary diffusion constants at those of free diffusion values (Figure 10A); and (3) the ability for the TEP to alter cluster characteristics by affecting the permeability of voltage-sensitive gap junctions (Figure 11C), inducing electroosmotic flows (Figure 11E), and inducing self-electrophoresis/electroosmosis of membrane-bound ion pumps and channels (Figure 11D) An additional simulation, whereby the TJ barrier was broken by removal of cells during the course of a simulation, demonstrated the role of tight junctions in creating a characteristic bioelectric signal with wounding (Figure 10C).

The mere presence of TJ, even in the absence of inhomogeneous membrane channel distribution, current, or electroosmotic flow, was seen to alter cell V_mem_ depending on a cell’s location with respect to the cluster boundary (Figures 10 and 11). Simulations showed a trans-boundary voltage difference of approximately 24 mV (analogous to the TEP observed in organs and organisms) spontaneously appeared across the inner and outer membranes of cluster boundary clusters when ion transport between extracellular spaces at the cluster boundary was limited by simulated TJ (Figure 11B shows the TEP as a close-up to the outer cell membranes). This trans-boundary voltage gradient did not appear when ion transport in extracellular spaces was similar to free-diffusion values for ions (Figure 10A), confirming the well-known role of TJ in establishing the TEP. As detailed in Figure 11A, tight junctions generate the TEP by maintaining a transport barrier at the cluster boundary, which acts analogously to the cell membrane to internalize charge emitted by cells, leading to the generation of a typically positive voltage in the extracellular spaces internal to the cluster at the exterior membranes. As the environmental space of apical cell membranes has zero voltage, there is a voltage difference between the apical and basal membranes of outer cells, which defines the TEP.

The wounding event transitioned the cluster into a new steady state with characteristic bioelectrical pattern. Cells local to the wounded area develop depolarized V_mem_ in addition to a characteristic pattern of current flow featuring current directed out of the wound, which returns back to the cluster at each side of the wound (Figure 10C). This well-known pattern of endogenous current flow and associated electric field are implicated in cellular signaling events for wound healing and the initiation of limb development (Borgens, 1984; Nuccitelli, 2003b; Zhao, 2009).

Finally, when TJ are present, the electric field generated by the TEP was predicted to influence voltage-sensitive gap junction permeability (Figure 11C) to spontaneously divide the cluster into two networks: an outer layer of GJ connected cells around the perimeter of the cluster, and the inner network of the cluster bulk, with the inner and outer layers isolated by low permeability gap junctions (Figure 11C). The TEP was also found capable of inducing electroosmotic flows with, on average, velocities of about 10 nm/s (Figure 11E) and to redistribute ion pump and channels in the membranes to generate polar cell fluxes across the apical and basal membranes (Figure 11D).

We conclude that the TEP, an important bioelectrical state characteristic of multicellular clusters, can arise spontaneously in cell clusters, simply by inhibiting diffusion from extracellular spaces of the cluster boundary. The TEP contributes to the generation of a characteristic cluster-wide bioelectric state upon wounding, and creates micro-environments with differential channel activity at the boundary and interior of the cluster.

#### 3.3.3 Simulation 8: Spontaneous V_mem_ Patterning

**Figure 12:**
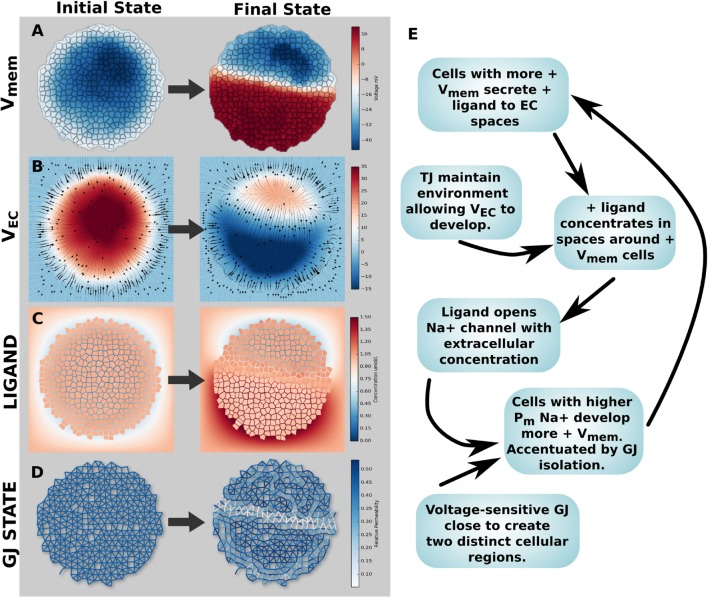
**Example feedback mechanism generating V_mem_ patterning in a cluster of voltage-sensitive, GJ networked cells with TJ, which occurs due to the presence of a charged channel gating ligand**. In its initial state, a cell cluster has a small V_mem_ asymmetry due to increase in K^+^ leak channels for a small cluster of cells in the upper right side, leading to a minor resting V_mem_ difference of approximately 10 mV [Initial State **(A)**]. This initial state also corresponds to small related asymmetries in environmental voltage [Initial State **(B)**], anionic ligand concentration [Initial State **(C)**], and no changes to GJ open/closed state [Initial State **(D)**, where blue = open, white = closed]. However, the presence of an electrodiffusing cationic gating ligand capable of opening a sodium channel based on its extracellular concentration (analogous to acetylcholine), in combination with TJ and GJ activity, generates a strong axial V_mem_ polarity on account of the positive feedback mechanism outlined in **(E)**.

A key question in this field is the origin of bioelectric pre-patterns. Turing and others (Turing, 1952; Cross and Hohenberg, 1993) showed that pattern can spontaneously self-organize from symmetry breaking in initially homogeneous media. Could bioelectric dynamics enable voltage pre-patterns to emerge spontaneously in physiologically homogeneous tissue? Simulation 8 explored spontaneous V_mem_ patterning (symmetry breaking) created by a positive feedback mechanism in the networked cell cluster. A wide range of feedback mechanisms exist in bioelectrical tissue systems on account of the chemical and V_mem_ sensitivity of ion channels and GJ, the non-linear relationship between channel/GJ activity and V_mem_, and the ability for voltage gradients to alter concentration profiles of ion channel gating ligands via electrodiffusive transport. Thus, we reasoned that positive feedback loops could amplify small physiological differences (noise) to result in macroscopic distributions that could underlie the origin of bioelectric pre-patterns. An example feedback mechanism was found capable of generating a strong, dipolar axial V_mem_ gradient in a cluster of voltage-sensitive GJ networked cells with TJ (Figure 12). In its initial state, the cluster had a small V_mem_ asymmetry of <10 mV, due to a small increase in K^+^ leak channels for a small number of cells in the upper right side (Figure 12A). This small asymmetrical expression of K + leak channels also corresponded to small related asymmetries in environmental voltage (Figure 12B) and anionic ligand concentration (Figure 12C). No changes to voltage-sensitive GJ open/closed state were noted in the initial state (Figure 12D, where blue = open, white = closed). However, the presence of an electrodiffusing cationic gating ligand, which opens a Na^+^ channel based on its extracellular concentration (a situation analogous to that of acetylcholine), in combination with TJ and GJ activity, generated a strong axial V_mem_ gradient (Figure 12).

The positive feedback loop is outlined in Figure 12E. TJ maintain an environment that internalizes charge, allowing a voltage to develop in extracellular spaces (Figure 12B), while cells with more depolarized V_mem_ exclude the cationic ligand to the extracellular spaces (Figure 12C). As voltage in the extracellular space is the inverse of that of intracellular spaces, the gating ligand also travels extracellularly from cells with more hyperpolarized to more depolarized cells (see currents in Figure 12B), leading to further build up of gating ligand around positive cells. Positive feedback results, as the positively charged gating ligand acts to open a Na^+^ ion channel, leading to cell V_mem_ becoming more depolarized. As voltage gradients develop between cells, GJ close, further reinforcing the development of two regions of highly distinguished V_mem_ (Figure 12B).

We conclude that it is possible for positive feedback mechanisms to generate strong V_mem_ gradients a cell collective, and that this may be one mechanism through which bioelectric pre-patterns may be generated and manipulated.

## 4 Discussion

Overall, BETSE enables highly detailed and accurate modeling of complex bioelectrical signals and states. A basic validation, using experimentally derived parameters, and comparing results with experimentally obtained observations for the same system (*Xenopus* oocytes), showed high correspondence (<10% discrepancy) between BETSE-calculated and experimental observables (Table 2). In addition, using the Nernst equation [equation (21)] to calculate V_mem_ on the basis of simulated electrodiffusing “reporter dye” intra- and extracellular concentrations, showed remarkable correspondence to BETSE direct-calculated V_mem_. Likewise, comparison between BETSE direct-calculated and Goldman-derived V_mem_ also showed excellent agreement. Multicellular simulations with TJ (Figure 10) also showed the characteristic TEP across the exterior cluster boundary, with typically observed polarity (inside positive) and magnitudes (~25 mV) close to those observed experimentally (Hay and Geddes, 1985). BETSE also correctly predicted the characteristic bioelectric signal occurring with wounding (Zhao, 2009) (Figure 10D). Simulated endogenous current flows with magnitudes from 1 to 200 *μ*A/cm^2^ were within the range of typically observed experimental currents 1–500 *μ*A/cm^2^, Nuccitelli (1992).

Dynamical system theory maintains the concept of an *attractor* as a parameter (or set of parameters) toward which a system tends to spontaneously evolve, even under a wide range of starting conditions (Milnor, 1985). An attractor state is also characterized by the system’s return to the attractor state after perturbation. Simulations 2 and 3 from our validation study emphasize how a cell’s resting V_mem_ is an attractor state that can be reached even with remarkably variable initial conditions, such as intracellular ion concentrations being equal to those of the extracellular environment, and starting voltages being zero (Figure 5; Table 3). Also consistent with the concept of an attractor state, even with transient perturbations, such as the introduction of a high K^+^ concentration to the environment, cell V_mem_ returns to its original resting value once the perturbation ceases (Figure 6). These properties are a powerful feature of bioelectric circuits, which implement robust and stable control elements during pattern formation under a wide range of conditions (McCaig et al., 2005; Levin, 2012).

Our validation simulations also demonstrate the expected timing and shape for excitable signals, which may become possible when cells express voltage-gated Na^+^ and K^+^ channels (Figure 7). Simulations with excitable channels also highlight the important influence resting V_mem_ has on cell excitability, with more depolarized resting potentials being able to induce periodic self-excitations (Figure 7), which is consistent with the mechanism of pacemaker cells of the heart. Some “somatic” cells, such as embryonic amphibian epithelium and some cancer cells, are known to express voltage-gated Na^+^ and K^+^ channels; due to their lower resting potential, action-potential-like signals, including self-excitations, are predicted by our model, and have also been observed experimentally (Roberts and Stirling, 1971; Onganer et al., 2005). Moreover, some aggressive metastatic cancers exhibit a depolarized resting V_mem_ with abnormal, high expression of voltage-gated Na^+^ and a deficient expression of voltage-gated K^+^ channels (Onganer et al., 2005; Djamgoz, 2014). Our results (Figure 7C) also indicate that low expression of voltage-gated K^+^, in combination with expression of voltage-gated Na^+^, may permanently alter resting V_mem_ from a hyperpolarized (−57 mV) to depolarized resting V_mem_ state (−14 mV) after a single transient depolarization event, which is consistent with the abnormal resting V_mem_ observed in some cancers (−20 to −5 mV) (Binggeli and Weinstein, 1986; Djamgoz, 2014). A similar form of resting V_mem_ bistability, also developing with the action of voltage-gated channels, has been described in the bioelectrical models of Cervera et al. (2014) and Law and Levin (2015).

Our studies also highlight the significant physiological impacts that resting V_mem_ can exert. One-way V_mem_ can exert an influence on downstream patterning mechanisms is by altering the spatial distribution of important chemical signaling molecules (such as Ca^2+^, serotonin, or glutamate) in relation to cell V_mem_, even when the compound is present at a homogeneous concentration in the extracellular bathing medium. This non-intuitive process happens because in an electrolyte medium where voltage gradients are present, ions are influenced, not only by concentration gradients (regular diffusion), but also by voltage gradients; under the right conditions they can passively move *up* concentration gradients. Thus, differences in V_mem_ alone can influence the cytosolic concentration of important signaling molecules, leading to differences in critical process, such as gene expression and enzyme function (Levin et al., 2006; Tseng et al., 2011). Our results indicate that the presence of regional V_mem_ differences can passively induce differences in cytosolic Ca^2+^ levels (Figure 8B) in a manner inversely related to cell V_mem_. As Ca^2+^ is an important secondary messenger, and intracellular Ca^2+^ levels are involved in calcium-induced-calcium-release (CICR) and ion channel gating (e.g., Ca^2+^ gated K^+^ channels), these differences in the cytosolic Ca^2+^ concentration with V_mem_ may produce significant downstream effects on cell state (including enzyme function, gene expression, and apoptosis) and further evolution of bioelectric pattern (via effects on Ca^2+^ gated ion channels). Similarly, effects on the concentration profile of a negatively charged signaling molecule were in direct correspondence to V_mem_ values (inverse to that of cationic Ca^2+^), which further illustrates how cell state can be influenced in divergent ways as by simply exhibiting different values of resting V_mem_ signaling molecules with different charge have opposite changes to their concentration profiles. Further V_mem_ related physiological changes, such as osmotic pressure gradients (Figure 8), can lead to differential changes in cell volume and the development of physical forces in the collective.

Due to the well demonstrated importance of cell resting V_mem_ states (McCaig et al., 2005; Tseng and Levin, 2013; Levin, 2014), clear comprehension of the factors involved in specifying cell resting V_mem_ is an essential first-step for understanding the mechanisms underlying bioelectrically mediated pattern formation and regulation. In single or isolated cells populating clusters without GJ or TJ, resting V_mem_ is primarily determined by the plasma membrane’s permeability to specific ions, to levels of extracellular ion concentration, and to the presence and activity of different ion pumps, such as H/K-ATPase (Veech et al., 1995; Lodish et al., 2000; Wright, 2004). BETSE accurately reproduces expected V_mem_ changes corresponding to these well-known factors of influence (Figures 5 and 6), with isolated cells showing strong depolarizations with increased Na^+^ membrane permeability, hyperpolarization with increased K^+^ permeability, depolarization with Cl^−^ membrane permeability, depolarization with increased extracellular K^+^, and hyperpolarization with H/K-ATPase pumps and moderate to high K^+^ membrane permeabilities (data not shown).

However, our studies also indicate that in cell clusters where bioelectric circuits are created via the presence of GJ (enabling intercellular communication) and TJ (blocking extracellular transport at the external boundary), factors influencing the resting V_mem_ attractor state can be quite different from those for isolated cells. Gap junction conductivity, TJ restriction at the boundary, and the overall geometry of the cluster are additional influences of the resting V_mem_ state that are unique to multicellular clusters. Simulations indicate that the degree of GJ connectivity has a strong affect on the ability for cells with altered membrane permeability properties (e.g., cells with a different ion channel expression profile or open ion channel state) to manifest different resting V_mem_ states in the collective (Figure 9). Since research indicates that resting V_mem_ is a key instructive signal (Levin and Stephenson, 2012; Pai et al., 2012), GJ are indicated as important elements determining how potent differential membrane ion channel states will be in affecting physiological outcomes. Our results are consistent with recent reports of Cervera et al. (2016) and Cervera et al. (2015), whose equivalent electrical circuit model of GJ connected cells demonstrates the wide range of cell V_mem_ that can result from differing GJ connectivity in a collective. It is also apparent that TJ (in conjunction with GJ) are responsible for the spontaneous emergence of a TEP, whereby cells at the outer boundary of a cell cluster acquire a depolarization compared with interior cells, thereby naturally forming a spatially dependent V_mem_ pattern.

The involvement of GJ- and TJ-related factors in the specification of multicellular V_mem_ attractor states sheds light on the importance of GJ and TJ in embryonic development, and help explain why loss of GJ permeability (Leithe et al., 2006) and increase in TJ permeability (Soler et al., 1999) are associated with cancer progression. With regard to embryonic development, our results suggest functional GJ and TJ play important roles in establishing primary patterns of V_mem_ and asymmetry in developing clusters (Figures 9 and 10). In cancer, which is characterized by cells with depolarized V_mem_, loss of GJ communication would allow cells with different membrane channel states to express dramatically altered V_mem_ compared to cells with high GJ connectivity (Figure 9), a conclusion that is also clearly shown by Cervera et al. (2016). Likewise, an increase in TJ permeability is predicted to result in decrease of the TEP with consequential depolarization of interior cells and atypical channel functions for cells on the boundary and interior (Figure 10). For the first time, BETSE allows quantitative study of tissue-level electric fields interacting with resting potential gradients – two key areas of developmental bioelectricity that have heretofore been studied separately.

Bioelectric circuits are rich with opportunities for feedback cycles, implying complex dynamics can exist in networks of connected cells, forming a layer of control with its own intrinsic behavior and self-organizing capabilities. Feedbacks exist because of non-linearities in the bioelectric system on a hierarchy of scales. On a fundamental level, voltages are created by net imbalances in ionic charge density; however, ionic concentrations are influenced by voltage gradients, thereby creating a primary non-linearity in the electrolytic system. Moving to the cellular and multicellular scale, the function of ion pumps and channels alters V_mem_, which in turn can affect voltage-sensitive channels and electrical synapses. Furthermore, the presence of ionic messengers, including Ca^2+^ or anionic serotonin or glutamate, are charged molecules subject to movement in electrochemical gradients, but can also alter V_mem_ directly via their effect on specific ion channels as gating ligands (Levin et al., 2006; Berridge, 2014). An example of a feedback mechanism capable of inducing a strong, axial V_mem_ gradient in a cell cluster was demonstrated by implementing dynamics similar to those of acetylcholine (Figure 12). It will be crucial to perform quantitative simulations of specific systems to understand the origin of the instructive bioelectric pre-patterns that regulate, for example, the formation of the vertebrate face (Vandenberg et al., 2011), and anterior–posterior polarity in planaria (Beane et al., 2013). As channelopathies are increasingly observed to be an important cause of birth defects (Bendahhou et al., 2003; Barel et al., 2008; Adams et al., 2016), such models will provide not only mechanistic explanations of the origin and progression of bioelectric pre-patterns but could also be used to test prospective interventions *in silico* for biomedical applications. Beyond embryogenesis, such quantitative models will reveal the conditions under which aberrant physiological states become normalized or established as tumors, and help formulate strategies for suppressing and perhaps reprograming established oncogenic states (Arcangeli et al., 2009; Chernet et al., 2015).

This first version of the BETSE platform has several limitations. First, BETSE currently considers a fixed number of cell grid points and does not compute cell division. Future work will extend the BETSE model to include the ability to model cell proliferation and apoptosis, as can occur downstream of bioelectric signaling in development, regeneration, and cancer. Similarly, cell mobility, including galvanotaxic movement of cells and galvanotropism in response to endogenous, global electric fields, is another key component of bioelectric pattern regulation. BETSE currently considers a fixed lattice of cells, which cannot move with respect to the environment. Cell and cluster shape changes in response to endogenous signals, such as the global current and field density are planned for future work. Besides the plasma membrane, intracellular organelles, such as the mitochondria, endoplasmic reticulum, and nucleus (Mazzanti et al., 2001), have their own bioelectic control mechanisms (subcellular membrane ion pumps and channels, as well as transmembrane voltages) which may interface with cell- and tissue-level bioelectric mechanisms. Models of these intracellular components are currently being developed for BETSE, in order to assist in understanding the hierarchy of interacting systems and sub-systems and their role in developmental/regenerative pattern and cancer development and regulation. Finally, we are working to add transcriptional readouts of bioelectric state change, to allow BETSE to model the control of gene expression by V_mem_ change and to integrate these models with existing *in silico* gene regulatory networks that underlie pattern regulation (Geard and Willadsen, 2009; Lobo and Levin, 2015).

Due to the complexity of bioelectric tissue systems, suitable and realistic models, such as BETSE are essential for exploring the properties and feedback mechanisms involved in bioelectrical circuits, and to elucidate the mechanisms involved in creating and regulating pattern. These tools represent an important enabling step for exploiting bioelectrical signaling in synthetic bioengineering approaches that harness self-organizing and control capabilities of voltage gradients for guided self-assembly of patterning tissues *in vitro*; moreover, they are a core component of forthcoming modeling tools that will identify specific manipulations of biophysical state that are predicted to achieve desired system-level outcomes (anatomical and physiological state). Future work will use BETSE to explore details of complex feedback loops involved in pattern emergence and dysregulation, with a focus on developing bioelectric interventions for rational control of morphogenesis (pattern emergence), regeneration (pattern after perturbation), and cancer development and suppression (pattern dysregulation).

## Author Contributions

ML and AP worked closely together on the functional spec and overall system strategy, based on project conceived by ML. AP designed and implemented the system, including all mathematical models, coding, and data representation. AP generated data from simulations, and analyzed it together with ML. AP and ML wrote the manuscript together.

## Conflict of Interest Statement

The authors declare that the research was conducted in the absence of any commercial or financial relationships that could be construed as a potential conflict of interest.
