# Supplementary material for: Exploring Instructive Physiological Signaling with the Bioelectric Tissue Simulation Engine
Source: Front Bioeng Biotechnol. 2016 Jul 6;4:55. doi: 10.3389/fbioe.2016.00055 (PMC4933718; doi:10.3389/fbioe.2016.00055)
Supplement: Supplementary file 1 [file Data_Sheet1.PDF]

Exploring Instructive Physiological Signaling with the Bioelectric Tissue Simulation Engine (BETSE)

Supplemental Theory and Implementation Details

Alexis Pietak and Michael Levin

Allen Discovery Center at Tufts University, Medford, MA, USA

BETSE (BioElectric Tissue Simulation Engine) is an open-source scientific software tool enabling bio-realistic modeling of dynamic electrochemical phenomena in gap junction-networked cell collectives, with a focus on the role of bioelectrics in spatio-temporal pattern formation.

The core methods of the BETSE model are finite volume techniques, which use discretized differential equations defined on grids with control volumes representing the cell and environmental architecture of the heterogeneous tissue. The following is a detailed walk-through of the BETSE model, covering computational, mathematical and theoretical aspects.

Contents

1 Model Overview . . . 2

2 Discrete Mathematics on BETSE Grids . . . 2

2.1 Overview of Grid Based Computations in BETSE . . . 2

2.2 The Cell Grid . . . 5

2.3 The Env Grid . . . 9

2.4 Mapping from cell to environment . . . 11

2.5 Types and definition of system variables . . . 11

2.6 Temporal dynamics . . . 13

2.7 Boundary and Initial conditions . . . 14

2.8 Assigning spatial properties using tissue profiles . . . 14

3 Membrane and Gap Junction Dynamics . . . 14

3.1 Hodgkin-Huxley model voltage gated channels 14
3.2 Gap junction voltage sensitivity 16
4 Additional Physical Mechanisms 17
4.1 Osmotic and hydrostatic pressure 17
4.2 Lateral movement of membrane pumps and channels 17
References 18

1 Model Overview

The system diagram of Figure 1 outlines the information flow paths used in BETSE to calculate various bioelectric system properties from ion concentrations in intra- and extracellular spaces. Not all of the functionality shown in Figure 1 is discussed in the present report.

2 Discrete Mathematics on BETSE Grids

2.1 Overview of Grid Based Computations in BETSE

Biological tissue represents a unique modeling scenario due to its highly heterogeneous nature, where closely-spaced (~10 to 30 nm) membrane bound, electrolyte-filled cells are individually interacting with a small extracellular space at individual plasma membranes, where the extracellular spaces connect with a continuous, aqueous environment at the cell cluster boundary. Individual cells are also connected internally via transmembrane channels such as gap junctions, which enable passage of small molecules between cells. BETSE uses an irregular Voronoi diagram based cell grid (*Cell Grid*) embedded within a regular square environmental grid (*Env Grid*) to model the heterogeneous nature of tissues, while also allowing modeling of a continuous environmental space around the cell cluster (Figure 2).

Finite volume methods define techniques by which differential equations can be discretized to be applied and solved on grids of points such as the *Cell Grid* and *Env Grid* of BETSE.

In general, two fundamentally different types of properties are *scalar properties*, such as concentration and voltage, which have a magnitude defined at each point in space, and *vector properties*, such as mass flux and electric field, which have both magnitude and direction at each point in space. For this discussion, scalar properties are represented abstractly as s_j – the hypothetical scalar property with magnitude s defined at grid-point j. Vector properties are represented as $\vec{F}_j$ with components F_{xj} and F_{yj} where $\vec{F}_j = F_{xj}\hat{x} + F_{yj}\hat{y}$. Here $\hat{x}$ and $\hat{y}$ are the unit vectors defining the horizontal and vertical directions of the space, respectively.

The core mathematical operators of differential equations used in BETSE are:

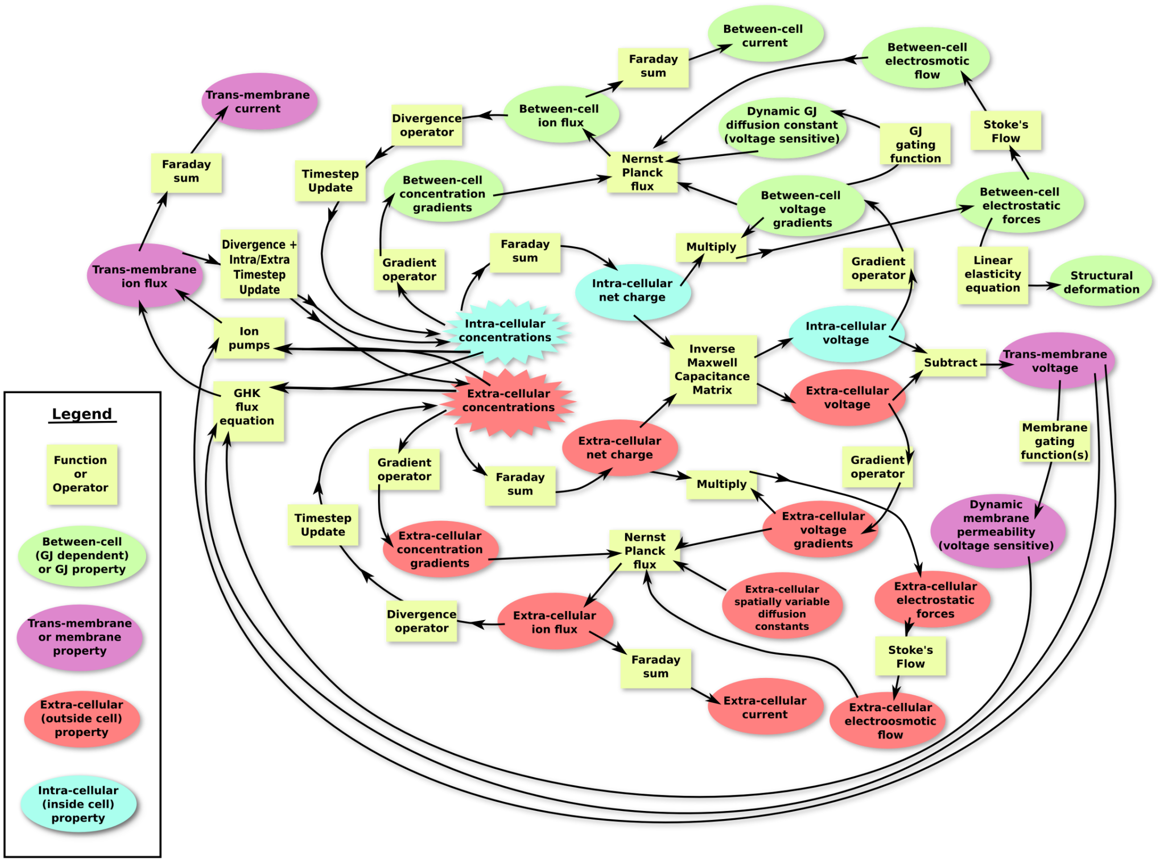

Fig. 1: System diagram detailing input/output relationships between BETSE variables and mathematical functions/operators occurring in a single time-step of a simulation.

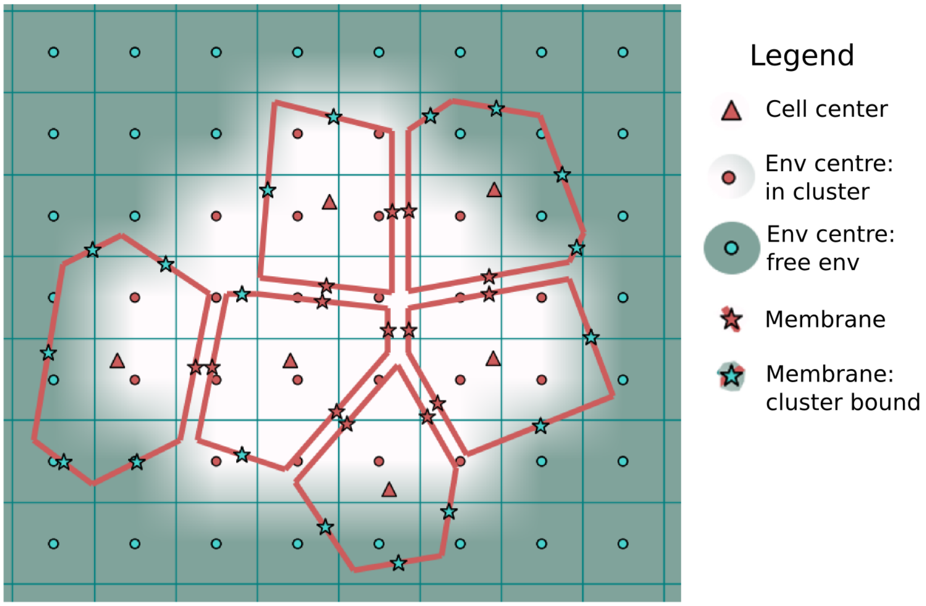

Fig. 2: BETSE computations use an irregular Voronoi-based *Cell Grid* embedded within a regular square *Env Grid* to model heterogeneous tissues composed of gap junction networked cells interacting with both a small extracellular space at the individual cell boundary, and a continuous environmental space at the cell cluster boundary. The *Cell Grid* is composed of cell center ('$\triangle$') and membrane points ('$*$'), with membranes lacking a neighboring cell (and therefore interacting with the global environment) identified. The *Env Grid* consists of regularly spaced points ('o') which are tagged as being internal (and interacting with a *Cell Grid* membrane point) or external to the cell cluster.

- *gradient* (∇s_j), which calculates the degree of change of the spatial property over space
- *divergence* ($\nabla \cdot \vec{F}_j$), which measures the amount of outward flow of a vector field from each point in space – the presence of a flux source, and
- the *Laplacian* ($\nabla^2 s_j = \nabla \cdot \nabla s_j$), which is most intuitively expressed as the divergence of the gradient of a scalar property. When discretized, the Laplacian is a matrix, which can be inverted to give the inverse of the operation (if $\nabla^2 S_j = c_j$ then $S_j = \nabla^{-2} c_j$)

Versions of gradient, divergence, and Laplacian/inverse Laplacian were defined, using first principles and finite difference/volume techniques, on the *Cell Grid* and *Env Grid*. These core mathematical operators were then used where required in specific differential equation expressions.

The sub-sections below describe the detailed features of the *Cell Grid* and *Env Grid,* and the specific definition of the three core mathematical operators.

2.2 The Cell Grid

Detailed features of the *Cell Grid* are illustrated in Figure 3. The *Cell Grid* was constructed from a Voronoi diagram built from a 2D scatter of seed points. The seed points become the center point of each cell control volume (cell centers indicated in grid diagrams as $\triangle$, see Figure 2), where scalar cell properties such as concentration (c_i) and intracellular voltage (V_{cell}) are defined.

Each cell has a unique volume (vol_{cell}) and perimeter, which were defined by scaling the vertices of each Voronoi patch in towards the cell center point. This allowed unique membrane properties, such as V_{mem}, as well as membrane-specific concentration (c_i) and intracellular voltage (V_{cell}), to be defined for each segment of each individual cell membrane. Membrane-specific scalar and vector properties are defined at each membrane midpoint. Membrane midpoints are indicated in some grid diagrams as $\star$, see Figure 2.

Volume vol_{cell} was calculated using the "shoelace formula" to obtain the area, A, of the individual cell region from its n vertices x_i, y_i :

$$A = \frac{1}{2}\left|\sum_{i=1}^{n-1}(x_i y_{i+1} + x_n y_1) - \sum_{i=1}^{n-1}(x_{i+1} y_i - x_1 y_n)\right| \quad (1)$$

and multiplying A by world height, h, which was arbitrarily selected to be $10\,\mu m$.

Each cell membrane segment:

- has a membrane midpoint coordinate point (x_{mem}, y_{mem}) labeled for one cell by the letters a to f in Figure 3A)

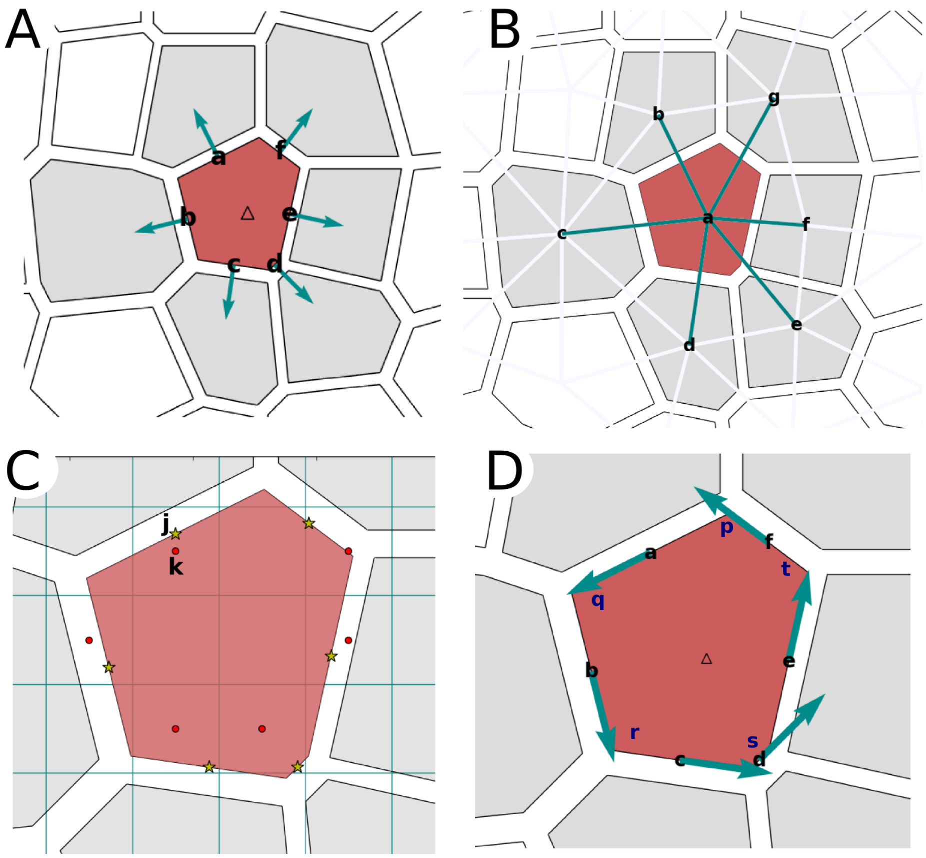

Fig. 3: Main features of the Cell Grid. Each cell is a region with unique volume and membrane segments with midpoints indicated by letters a to f in Panel A. Each cell connects to neighboring cells via a gap junction connecting two opposing membranes (green lines Panel B). Each membrane segment has normal and tangent unit vectors (A and D, respectively). The membrane midpoints of each cell (yellow stars Panel C) interface with the central points of local *Env Grid* squares (red points Panel C) via a nearest-neighbor interpolation scheme (j, k of Panel C shows a specific *Env Grid* to *Cell Grid* pairing).

- has a surface area σ_{mem}, defined by multiplying the cell side segment length by h,
- has surface normal $\hat{n}_{mem_x}$, $\hat{n}_{mem_y}$ (Figure 3A) and tangent vectors $\hat{t}_{mem_x}$, $\hat{t}_{mem_y}$ (Figure 3D)
- connects to a neighboring cell with a membrane segment of the same length/area via a gap junction (Figure 3B).
- interfaces with the points of the *Env Grid* via a nearest-neighbor interpolation scheme (Figure 3C).

Gradients on the *Cell Grid*

Three types of gradients can be calculated on the *Cell Grid*:

1. *Inter-cellular* gradients calculate change of a scalar property, such as concentration or voltage, between cells and their neighbors. For a scalar property s defined on neighboring cell centers a and b (Figure 3B) separated by distance d_{ab} :

$$F_{ab} = \frac{(s_b - s_a)}{d_{ab}} \tag{2}$$

The $x-$ and $y-$ components of the gradient are found by resolving F_{ab}into the gap junction tangent vector components, and are defined at the midpoint (ab) between the neighboring cells:

$$\nabla s_{ab} = F_{ab}\,\hat{t}_{gj_x} + F_{ab}\,\hat{t}_{gj_y} \tag{3}$$

Note that for gap junction specific gradient calculations (e.g. the electric field across a gap junction) the distance d_{ab} is taken to be the biological intercellular separation/gap junction length of $d_{gj} = 26nm$, instead of the cell-cell center spacing of the *Cell Grid*. As the intercellular gradient is based on the network properties of the *Cell Grid* via existing gap junction connections (3 B), the boundary conditions for the inter-cellular gradient always enforce zero-flux at the cluster boundary.

2. *Trans-membrane* gradients calculate change of a scalar property, such as concentration or voltage, between a cell and its immediate environment (Figure 3C). For a scalar property s defined on environmental point j and membrane point k, the transmembrane gradient is calculated with respect the membrane thickness d_{mem} as:

$$\nabla s_{jk} = \frac{(s_j - s_k)}{d_{mem}} \tag{4}$$

The transmembrane gradient is assumed to only have a component in the direction perpendicular to the cell membrane normal.

3. *Intra-membrane* gradients calculate lateral (i.e. tangential) change of a scalar property, such as the concentration of an ion channel, between points of an individual cell's membrane. For a scalar property s defined on cell vertices p and q:

$$\nabla s_{pq} = \frac{(s_q - s_p)}{d_{pq}} \tag{5}$$

The separation between p and q is the membrane length. The gradient vector, which is assumed to only have a tangential component, is defined at the membrane midpoint between the two vertices (see Figure 3D).

Divergence on the *Cell Grid*

Divergence is a scalar property defined at the cell center points for the *Cell Grid* using each individual cell patch as a control volume. By definition, the divergence of a vector field $\vec{F}$

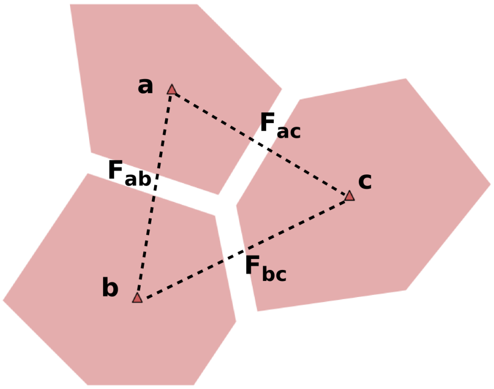

Fig. 4: Laplacian calculation for a simple cell grid.

at a point j is the limit of the net flow of $\vec{F}$ across the boundary of a region surrounding j, divided by the volume of the region, in the limit that the volume shrinks to an infinitesimal value around j.

This formal definition was used to approximate divergence on the *Cell Grid*. For a vector property $\vec{F}_j$ defined at each membrane midpoint j of an individual cell k, components of the vector property normal to the cell membrane ($\vec{F}_j \cdot \hat{n}_{mem_j}$) were multiplied by the membrane surface area $\sigma_{mem_j}$to obtain an approximation of net flux across the membrane, and all net fluxes were summed for each membrane of the cell, with the result divided by cell volume (see Figure 3A). The resulting divergence property is scalar and defined on the cell k center point:

$$\nabla \cdot \vec{F}_k = \frac{\sum \vec{F}_j \cdot \hat{n}_{mem_j} \sigma_{mem_j}}{vol_{cell_k}} \tag{6}$$

This method is identical to the finite volume approximation of divergence using the Divergence Theorem definition (Schafer 2006), with the assumption that the vector field is constant within the cell region. Physically, this assumes no concentration or voltage gradients exist within the cell (homogeneous concentration and charge distributions).

Laplacian (and inverse) on the *Cell Grid*

The Laplacian operator can be expressed as the divergence of the gradient of a scalar property. This definition was used to construct a matrix-based Laplacian operator and inverse operator for the cell grid.

For a scalar property s defined on three cells a, b, and c, which are assumed connected to one another but closed from the environment, the calculation of the Laplacian of s at cell a begins by calculating the gradient fluxes, $F_{ab}$and F_{ac} , of s between cell a and its neighbors (see Figure 4). Next, the normal components of these fluxes to the membranes are obtained using the membrane normal unit vectors (e.g. $\hat{n}_{x_{ab}}$ and $\hat{n}_{y_{ab}}$), and net flux is obtained by multiplying the flux by the membrane surface area of the shared membrane (e.g. σ_{ab}):

$$\begin{aligned} \vec{F}_{ab} \cdot \hat{n}_{ac}\, \sigma_{ab} &= \left(\frac{s_b - s_a}{d_{ab}}\right) \sigma_{ab}\, \hat{n}_{x_{ab}} + \left(\frac{s_b - s_a}{d_{ab}}\right) \sigma_{ab}\, \hat{n}_{y_{ab}} \\ \vec{F}_{ac} \cdot \hat{n}_{ac}\, \sigma_{ac} &= \left(\frac{s_c - s_a}{d_{ac}}\right) \sigma_{ac}\, \hat{n}_{x_{ac}} + \left(\frac{s_c - s_a}{d_{ac}}\right) \sigma_{ac}\, \hat{n}_{y_{ac}} \end{aligned} \tag{7}$$

The resulting divergence of the property s at cell a is the sum of the components from equation (7), divided by cell a volume:

$$\begin{aligned} \nabla^2 s_a &= A\, s_a + B\, s_b + C\, s_c \\ A &= \frac{-2\,\sigma_{ab}\,\left(\hat{n}_{x_{ab}} + \hat{n}_{y_{ab}}\right)\, d_{ac} - 2\,\sigma_{ac}\,\left(\hat{n}_{x_{ac}} + \hat{n}_{y_{ac}}\right)\, d_{ab}}{d_{ac}\, d_{ab}\, vol_a} \\ B &= \frac{\sigma_{ab}\,\left(\hat{n}_{x_{ab}} + \hat{n}_{y_{ab}}\right)}{d_{ab}\, vol_a} \\ C &= \frac{\sigma_{ac}\,\left(\hat{n}_{x_{ac}} + \hat{n}_{y_{ac}}\right)}{d_{ac}\, vol_a} \end{aligned} \tag{8}$$

The ability to express the Laplacian for each cell as an expression factored with respect to the cell-centered property s_j allows for the definition of a Laplacian operator matrix, $\mathbf{M_{Lap}}$, which can be matrix-multiplied with a linear vector of concentrations defined on cell points (e.g. $\mathbf{s} = [s_a, s_b, s_c]$) to perform the Laplacian computation. For the simple example above, and only considering cell a components, where A, B and C are the terms defined in equation 8 above and / represents a term in the matrix that this discussion has not defined:

$$\nabla^2 s = \mathbf{M_{Lap}} \cdot \mathbf{s} = \begin{matrix} A & B & C \\ / & / & / \\ / & / & / \end{matrix} \quad \begin{matrix} s_a \\ s_b \\ s_c \end{matrix} \tag{9}$$

The inverse Laplacian is easily calculated from the Laplacian defined in equation 9 by taking the pseudo-inverse using the singular value decomposition method in the 'pinv' function of the Numpy toolbox.

2.3 The Env Grid

The environmental grid consists of a regular array of center points spaced by d_{grid} (~ 5 μm). In addition to the center points, the *Env Grid* uses a Marker and Cells (MACs) technique

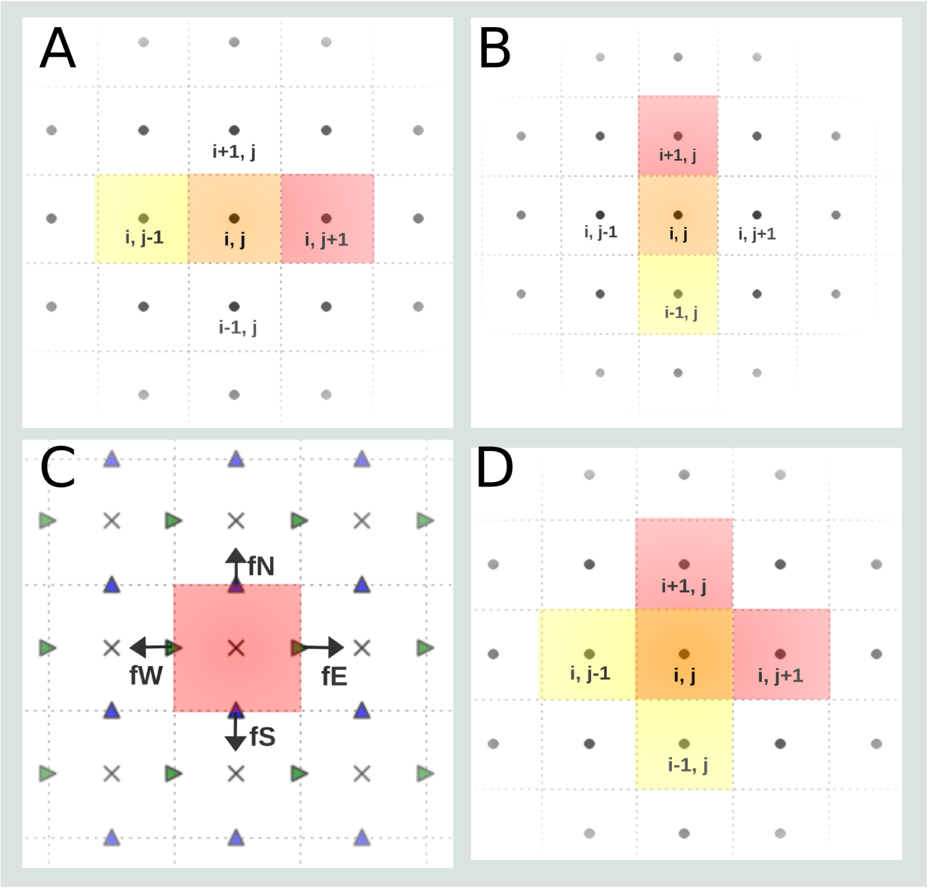

Fig. 5: The *Env Grid* consists of regularly spaced square centers. Calculation of simple x and y gradients using a central difference scheme is schematically illustrated in Panels A and B. Two additional staggered arrays define points at the W, E and N, S sides of each grid square (Panel C), where vector properties are defined. Panel D schematically illustrates the stencil used to obtain a Laplacian operator.

(McKee et al. 2008) which features two staggered grids defining W, E points on the sides of each square, and N, S points on the top and bottom of each square (see Figure 5C). Vector properties are defined with F_x components on the W and E sides of each grid square (Figure 5), and F_y components on the N and S sides of each square (Figure 5). The MACs method was found to improve computational stability for the non-linear electrodiffusion of concentrations in the environment.

Gradient, divergence, and Laplacian (and inverse Laplacian) discrete operators were defined using a similar approach to that described for the *Cell Grid*.

Gradients on the *Env Grid*

First derivatives in space were estimated using the central difference formula for a grid with uniform spacing d_{grid} and points on the grid indexed by their j^{th} row and k^{th} column (see

Figure 5A and B):

$$\frac{ds(x_j, y_i)}{dx} \Rightarrow \frac{s(x_{j+1}, y_i) - s(x_{j-1}, y_i)}{2d_{grid}} \tag{10}$$

$$\frac{ds(x, y)}{dy} \Rightarrow \frac{s(x_j, y_{i+1}) - s(x_j, y_{i-1})}{2d_{grid}} \tag{11}$$

Derivatives use center points of the grid squares ('x' markers in Figure 5C), while the x and y components of the resulting gradient are defined on the grid sides, as described above and shown in (Figure 5C).

Divergence on the *Env Grid*

Divergence on the Env Grid was handled using a method similar to that described for the cell grid, also see (McKee et al. 2008).

Laplacian on the *Env Grid*

The discrete Laplacian was calculated using the definition of the Laplacian as the divergence of the gradient of a scalar process, using a similar process to that defined for the *Cell Grid* in equations 8 and 9.

$$\nabla^2 s(x, y) = \left(\frac{s_{i+1,j} - s_{ij}}{d_{grid}} + \frac{s_{i-1,j} - s_{ij}}{d_{grid}} + \frac{s_{i,j+1} - s_{ij}}{d_{grid}} + \frac{s_{i,j-1} - s_{ij}}{d_{grid}} \right) \tag{12}$$

2.4 Mapping from cell to environment

The Cell Grid and Env Grid variables were connected by mass fluxes between the two environments, as well as via the transmembrane voltage V_{mem}. Fluxes and V_{mem} were calculated using gradients between pairs of points representing a cell membrane and its nearest environmental grid square central point (Figure 3C). A weighting function (cell membranes seen per grid square) was used to properly assign the mole transfer for a mass flux between cell and environment (Figure 6).

2.5 Types and definition of system variables

The following section outlines how specific properties are defined on BETSE's Cell Grid and Env Grid.

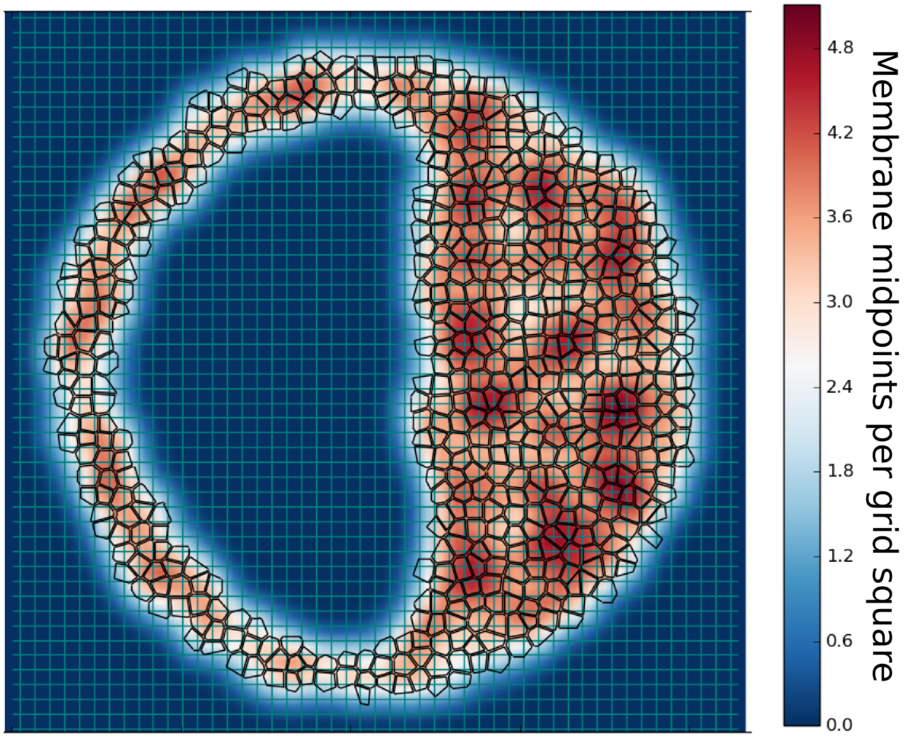

Fig. 6: The intracellular spaces of the the Cell Grid are assumed to be connected to the extracellular environment by fluxes or gradients, which use points interpolated between the cell membrane and environmental grid midpoints with a weighting function (cell membranes seen per grid square) to properly assign the mole transfer for a mass flux between cell and environment.

Cell Grid

*Concentration, charge, and voltage; scalar propert*ies. Concentrations of ions and other substances, intracellular voltage, and intracellular charge are scalar properties defined on cell center points and membrane midpoints (Figure 3). Ion concentrations are subjected to diffusion within individual cells.

Transmembrane Voltage (V_{mem}); scalar property. V_{mem} is defined on each cell's membrane midpoint (Figure 3), as the difference between local intracellular and extracellular voltages ($V_{mem_i} = V_{cell_i} - V_{env_i}$). This allows for a single cell to have different V_{mem} at difference regions around its circumference, which enables study of single-cell membrane polarizations.

Flux, current, field, velocity; vector properties. Ion flux, ion current, electric field, and fluid velocities are defined at each membrane midpoint (Figure 3), and are assumed to be normal to the membrane boundary for both transmembrane ion flux and intercellular membrane flux.

Ion pump/channel concentration (scalar) and flux (vector). Membrane-bound ion pump and channel concentrations are defined at each membrane midpoint (Figure 3).

Env Grid

*Concentration, charge, and voltage; scalar propert*ies. Concentrations of ions and other substances, extracellular voltage, and extracellular charge are scalar properties defined on the center points of each *Env Grid* square (Figure 5). These properties are assumed constant throughout each square of the grid.

Flux, current, field, velocity; vector properties. Ion flux, ion current, electric field, and fluid velocities are defined at midpoints between two grid square centers (Figure 5). Vector properties have F_x components defined on the W and E sides of each grid square (Figure 5) and F_y components on the N and S sides of each square (Figure 5).

2.6 Temporal dynamics

Time dependent properties $f(x, y, t)$ were updated with respect to changes in time using Euler's method for a time-step Δt:

$$f(x, y, t_{k+1}) = f(x, y, t_k) + \Delta t \, g(x, y, t_k) \tag{13}$$

Where $g(x, y, t_k)$ is the estimated rate of change of $f(x, y, t_k)$ at the discrete time point t_k.

2.7 Boundary and Initial conditions

Boundary conditions were set at the global boundary of the *Env Grid* to be zero voltage and fixed concentration of all ions at the global boundary, whereby the sum of the fixed concentration set yielded zero net charge (bulk electro-neutrality of the electrolyte).

The concentrations or voltage at the global boundary can optionally be changed during the course of a simulation to simulate the addition of a reagent (e.g. excess KCl) or application of an external voltage to the cell cluster.

For typical simulations, the system begins with an initialization phase with zero charge and zero voltage in cells and environment. Concentration profiles inside and outside of the cell typically begin close to those found physiologically. After an initialization phase of approximately 10 simulated seconds, the simulation begins using initial concentration and voltage conditions obtained from the completed initialization phase.

2.8 Assigning spatial properties using tissue profiles

The cell grid can be shaped using bitmaps as masks to selectively remove cell patches from the *Cell Grid* (Figure 7). The *Cell Grid* can be shaped prior to a simulation to generate a specific tissue shape. Alternatively cells can be removed during a simulation to simulate a wounding process.

Bitmap masks are also used to define tissue profiles, to which different properties (e.g. membrane permeability, presence of specific ion channel types) can be assigned to cells and their membranes.

3 Membrane and Gap Junction Dynamics

3.1 Hodgkin-Huxley model voltage gated channels

A range of voltage gated channel types have been implemented in BETSE using Hodgin-Huxley style differential equations to define the state of membrane diffusion to a specific ion (e.g. Na^{+}) as a function of V_{mem} and time. Specific parameters and functional relations were obtained from the online database, *Channelpedia* (Ranjan et al. 2011).

The present work specifically uses a combined generic voltage gated sodium channel (NaV) from (Hamill et al. 1991), and a delayed-rectifier voltage gated potassium channel (KV1.2) from (Sprunger et al. 1996), to generate excitable signals. A standard Hodgkin-Huxley style model uses an electrical equivalent circuit equation to determine changes to current and voltage across a membrane, with a set of differential equations controlling the conductance of the membrane (Nelson 2004). Since conductance is proportional to the membrane diffusion

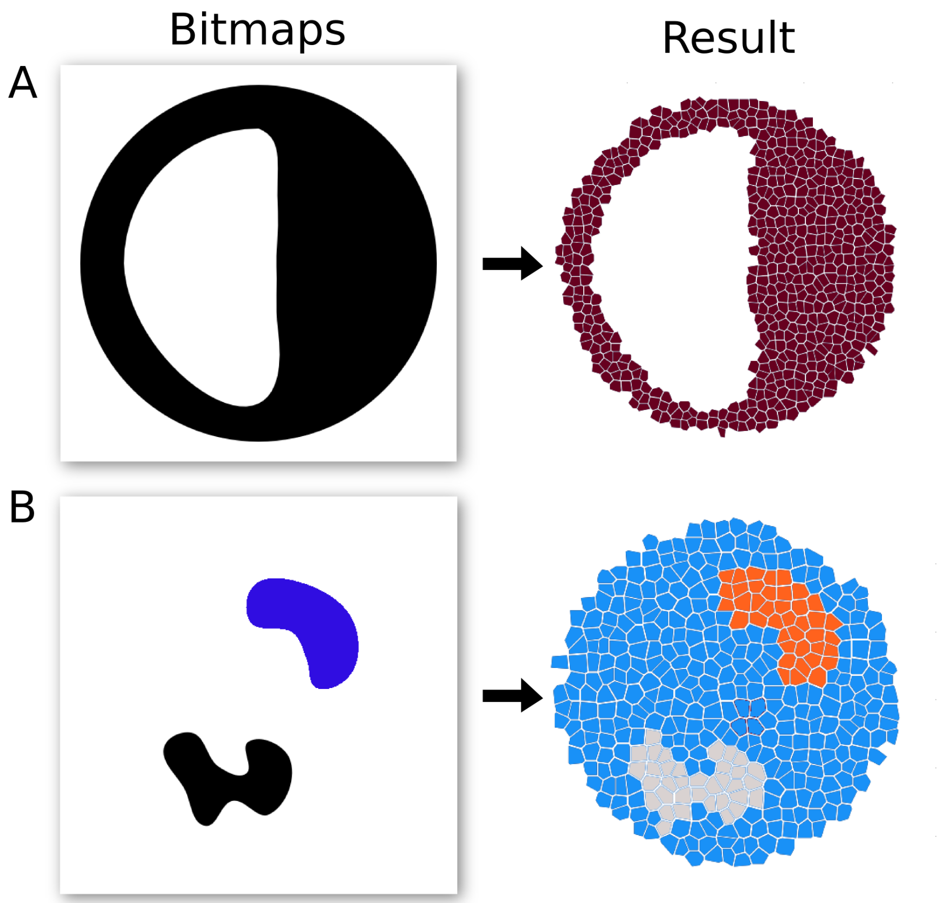

Fig. 7: Bitmap masks can be used to shape the overall cell cluster (A) and to define regions where properties, such as membrane permeability or the presence of a dynamic ion channel, can be easily assigned (B).

constant for a particular ion, BETSE uses the same Hodgkin-Huxley style equations developed to describe membrane conductivity state to describe the membrane diffusion state of a particular ion, updating subsequent changes to currents and voltages using its own methods, as described in the above.

As an example, for the generic NaV channel model obtained from Hamill et al. 1991, the dynamic NaV contribution to a cell's sodium membrane permeability $D_{mem_{Na}}$ would be expressed:

$$D_{mem_{Na}} = D_{NaV}(m^3 h) \tag{14}$$

Where D_{NaV} is the maximum membrane diffusion to Na^+ when the NaV channel is completely open and:

$$\begin{aligned} \frac{dm}{dt} &= (m_\infty - m)/m_\tau \\ \frac{dh}{dt} &= (h_\infty - h)/h_\tau \end{aligned} \tag{15}$$

with:

$$\begin{aligned} m_\alpha &= 0.182\left(\frac{(V_{mem}+25)}{1-exp(-\frac{(v+25)}{9})}\right) \\ m_\beta &= 0.124\left(\frac{-(V_{mem}+25)}{1-exp(\frac{(v+25)}{9})}\right) \\ m_\infty &= \frac{m_\alpha}{(m_\alpha+m_\beta)} \\ m_\tau &= \frac{1}{(m_\alpha+m_\beta)} \\ h_\infty &= \frac{1}{1+exp(\frac{(V_{mem}+55)}{6.2})} \\ h_\tau &= \frac{1}{0.024\,(V_{mem}+40)}\,(1-exp(-(V_{mem}+40)/5)) + \left(\frac{0.0091\,(-V_{mem}-65.0)}{1-exp(V_{mem}+65.0)}\right)5 \end{aligned} \tag{16}$$

3.2 Gap junction voltage sensitivity

Gap junctions were modeled as (optionally) voltage-sensitive conduits influencing the intercellular diffusion coefficient for all ions uniformly via a diffusion-constant scaling factor, β^o_{GJ}. Simulated transport through GJ used the Nernst-Planck Equation to update concentration of

all ions moving under intercellular concentration and voltage gradients. In the absence of GJ, cells were modeled to have an intercellular diffusion coefficient of zero ($\beta^o_{GJ} = 0$). Medium-high GJ connectivity corresponded to β^o_{GJ}=1.0x10^{-6}, an intercellular diffusion coefficient of approximately 1.0x10^{-15}m^2/s. Assuming 1.0x10^5 GJ per cell, and cylindrical GJ with pore diameter of 1.5 nm and length of 26 nm, this corresponds to individual GJ conductance of 68 pS, which is in the mid-range of reported GJ conductances (Goodenough and Paul 2009).

Voltage gating of GJ was described using the kinetic model of Harris et al. 1983, which calculates GJ open/closed state (β_{GJ}) dependence on voltage difference across the gap junction (V_{GJ}) and time via:

$$\begin{aligned} g_{min} &= 0.04 \\ m_{GJ} &= 0.0013\, exp(-0.077\,(V_{GJ} - v_{1/2GJ})) \\ n_{GJ} &= 0.0013\, exp(0.14\,(V_{GJ} - v_{1/2GJ})) \\ \frac{dP_{GJ}}{dt} &= (1 - P_{GJ})\, m_{GJ}\, V_{GJ} - (P_{GJ} - g_{min})\, n_{GJ}\, V_{GJ} \\ \beta_{GJ} &= \beta^o_{GJ}\, P_{GJ} \end{aligned} \tag{17}$$

4 Additional Physical Mechanisms

4.1 Osmotic and hydrostatic pressure

The osmotic pressure gradient across a cell membrane was estimated using the Jacobus van't Hoff formula, subtracting osmotic pressure inside the cell from the pressure outside of the cell.

$$\triangle \prod = \left(\sum c_{cell_i} - \sum c_{env_i}\right)\, R\, T \tag{18}$$

4.2 Lateral movement of membrane pumps and channels

Lateral movements of membrane pumps and channels were calculated using the gradients of concentration and voltage tangent to Cell Grid membrane segments, as well as the tangential component of environmental electroosmotic flow at the boundary.

Fluxes were calculated by averaging membrane midpoint values of a scalar property to the vertices. Next, gradients on the membrane were calculated between each vertex point p and r around each cell, with d_{pr} representing the membrane length and the membrane midpoint being q. For instance, for a concentration c of ion pumps, the gradient along a membrane segment is defined as:

$$\nabla c_q = \frac{(c_r - c_p)}{d_{rp}} \tag{19}$$

The flux from the Nernst-Planck equation Φ_q was calculated from concentrations, tangential fluid velocity $u_t$and tangential electric field E_t via:

$$\Phi_q = -\nabla c_q + u_t c_q - \left(\frac{D\, z\, q\, c_q}{k_b\, T}\right) E_t \tag{20}$$

Using the derivative definition of divergence:

$$\nabla \cdot \Phi = \frac{\partial \Phi_x}{\partial x} + \frac{\partial \Phi_y}{\partial y} \tag{21}$$

The x, y components of the flux were first resolved using the membrane tangent vectors:

$$\begin{aligned} \Phi_{qx} &= \Phi_q\, \hat{t}_x \\ \Phi_{qy} &= \Phi_q\, \hat{t}_y \end{aligned} \tag{22}$$

These flux components were averaged to the cell vertices. The derivatives of the flux components were calculated as:

$$\begin{aligned} \frac{\partial \Phi_x}{\partial x} &= \frac{(\Phi_{rx} - \Phi_{px})}{d_{rp}}\, \hat{t_x} \\ \frac{\partial \Phi_y}{\partial y} &= \frac{(\Phi_{ry} - \Phi_{py})}{d_{rp}}\, \hat{t_y} \end{aligned} \tag{23}$$

The concentration was updated according to:

$$\frac{\partial c_q}{\partial t} = -\nabla \cdot \Phi_q = -\frac{(\Phi_{rx} - \Phi_{px})}{d_{rp}}\, \hat{t_x} - \frac{(\Phi_{ry} - \Phi_{py})}{d_{rp}}\, \hat{t_y} \tag{24}$$

References

Goodenough, D. and Paul, D. (2009). Gap junctions. *Cold Spring Harbour Perspectives on Biology* 1, a002576

Hamill, O., Huguenard, J., and Prince, D. (1991). Patch-clamp studies of voltage-gated currents in identified neurons of the rat cerebral cortex. *Cerebral Cortex* 1, 48–61

Harris, A., Spray, D., and Bennett, M. (1983). Control of intercellular communication by voltage dependence of gap junctional conductance. *The Journal of Neuroscience* 3, 79–100

McKee, S., Tome, M., Ferreira, V., Cuminato, J., Castelo, A., Sousa, F., et al. (2008). The mac method. *Computers and Fluids* 37, 907–930

Nelson, M. (2004). *Databasing the Brain: From Data to Knowledge* (Wiley, New York), chap. Electrophysiological Models

Ranjan, R., Khazen, G., Gambazzi, L., Ramaswamy, S., Hill, S., Schürmann, F., et al. (2011). Channelpedia: an integrative and interactive database for ion channels. *Fronteirs in Neuroscience* 5, 1–8

Schafer, M. (2006). *Computational Engineering – Introduction to Numerical Methods*, chap. 4: Finite volume methods. 77–105

Sprunger, L., Stewig, N., and O'Grady, S. (1996). Effects of charybdotoxin on k+ channel (kv1.2) deactivation and inactivation kinetics. *European Journal of Pharmacology* 314, 357–364
